# A combination of urinary biomarker panel and PancRISK score for earlier detection of pancreatic cancer: A case–control study

**DOI:** 10.1371/journal.pmed.1003489

**Published:** 2020-12-10

**Authors:** Silvana Debernardi, Harrison O’Brien, Asma S. Algahmdi, Nuria Malats, Grant D. Stewart, Marija Plješa-Ercegovac, Eithne Costello, William Greenhalf, Amina Saad, Rhiannon Roberts, Alexander Ney, Stephen P. Pereira, Hemant M. Kocher, Stephen Duffy, Oleg Blyuss, Tatjana Crnogorac-Jurcevic

**Affiliations:** 1 Centre for Cancer Biomarkers and Biotherapeutics, Barts Cancer Institute, Queen Mary University of London, London, United Kingdom; 2 Centro Nacional de Investigaciones Oncológicas, Madrid, Spain; 3 Centro de Investigación Biomédica en Red de Cáncer, Madrid Spain; 4 Department of Surgery, University of Cambridge, Cambridge, United Kingdom; 5 Institute of Medical and Clinical Biochemistry, Faculty of Medicine, University of Belgrade, Belgrade, Serbia; 6 Molecular and Clinical Cancer Medicine, University of Liverpool, Liverpool, United Kingdom; 7 Centre for Tumour Biology, Barts Cancer Institute, Queen Mary University of London, London, United Kingdom; 8 Institute for Liver and Digestive Health, University College London, London, United Kingdom; 9 Centre for Cancer Prevention, Wolfson Institute of Preventive Medicine, Queen Mary University of London, London, United Kingdom; 10 School of Physics, Astronomy and Mathematics, University of Hertfordshire, Hatfield, United Kingdom; 11 Department of Paediatrics and Paediatric Infectious Diseases, Institute of Child Health, Sechenov First Moscow State Medical University, Moscow, Russia; 12 Department of Applied Mathematics, Lobachevsky State University of Nizhny Novgorod, Nizhny Novgorod, Russia; Indiana University School of Medicine, UNITED STATES

## Abstract

**Background:**

Pancreatic ductal adenocarcinoma (PDAC) is one of the deadliest cancers, with around 9% of patients surviving >5 years. Asymptomatic in its initial stages, PDAC is mostly diagnosed late, when already a locally advanced or metastatic disease, as there are no useful biomarkers for detection in its early stages, when surgery can be curative. We have previously described a promising biomarker panel (LYVE1, REG1A, and TFF1) for earlier detection of PDAC in urine. Here, we aimed to establish the accuracy of an improved panel, including REG1B instead of REG1A, and an algorithm for data interpretation, the PancRISK score, in additional retrospectively collected urine specimens. We also assessed the complementarity of this panel with CA19-9 and explored the daily variation and stability of the biomarkers and their performance in common urinary tract cancers.

**Methods and findings:**

Clinical specimens were obtained from multiple centres: Barts Pancreas Tissue Bank, University College London, University of Liverpool, Spanish National Cancer Research Center, Cambridge University Hospital, and University of Belgrade. The biomarker panel was assayed on 590 urine specimens: 183 control samples, 208 benign hepatobiliary disease samples (of which 119 were chronic pancreatitis), and 199 PDAC samples (102 stage I–II and 97 stage III–IV); 50.7% were from female individuals. PDAC samples were collected from patients before treatment. The samples were assayed using commercially available ELISAs. Statistical analyses were performed using non-parametric Kruskal–Wallis tests adjusted for multiple comparisons, and multiple logistic regression. Training and validation datasets for controls and PDAC samples were obtained after random division of the whole available dataset in a 1:1 ratio. The substitution of REG1A with REG1B enhanced the performance of the panel to detect resectable PDAC. In a comparison of controls and PDAC stage I–II samples, the areas under the receiver operating characteristic curve (AUCs) increased from 0.900 (95% CI 0.843–0.957) and 0.926 (95% CI 0.843–1.000) in the training (50% of the dataset) and validation sets, respectively, to 0.936 in both the training (95% CI 0.903–0.969) and the validation (95% CI 0.888–0.984) datasets for the new panel including REG1B. This improved panel showed both sensitivity (SN) and specificity (SP) to be >85%. Plasma CA19-9 enhanced the performance of this panel in discriminating PDAC I–II patients from controls, with AUC = 0.992 (95% CI 0.983–1.000), SN = 0.963 (95% CI 0.913–1.000), and SP = 0.967 (95% CI 0.924–1.000). We demonstrate that the biomarkers do not show significant daily variation, and that they are stable for up to 5 days at room temperature. The main limitation of our study is the low number of stage I–IIA PDAC samples (*n* = 27) and lack of samples from individuals with hereditary predisposition to PDAC, for which specimens collected from control individuals were used as a proxy.

**Conclusions:**

We have successfully validated our urinary biomarker panel, which was improved by substituting REG1A with REG1B. At a pre-selected cutoff of >80% SN and SP for the affiliated PancRISK score, we demonstrate a clinically applicable risk stratification tool with a binary output for risk of developing PDAC (‘elevated’ or ‘normal’). PancRISK provides a step towards precision surveillance for PDAC patients, which we will test in a prospective clinical study, UroPanc.

## Abstract 2

Silvana Debernardi and colleagues establish a clinical risk score and a biomarker panel for early detection of pancreatic cancer.

## Author summary

**Why was this study done?:**

- Pancreatic ductal adenocarcinoma (PDAC) is one of the most lethal cancers, with around 9% of individuals surviving more than 5 years.
- The poor outcome is largely due to late diagnosis; however, if the disease is detected at an early stage, when tumours are still small and resectable, 5-year survival can increase to up to 70%.
- The development of a non-invasive test for the early detection of PDAC is therefore a major clinical need, likely to improve the chances of patients’ survival.

**What did the researchers do and find?:**

- We have previously identified a panel of 3 protein biomarkers (LYVE1, REG1A, and TFF1) in urine that showed promise in detecting resectable PDAC.
- In this study, we have improved this panel by substituting REG1A with REG1B, validated this panel in an additional number of retrospectively collected samples, and shown the ability of our urinary panel to distinguish control individuals and patients with benign hepatobiliary diseases from early stage PDAC patients with specificity and sensitivity >85%.
- Different cutoffs were tested for the PancRISK score, an algorithm that enables the stratification of patients into those with ‘normal’ or ‘elevated’ risk for developing PDAC: PancRISK-Fam can be useful for surveillance of asymptomatic individuals with genetic predisposition for PDAC, and PancRISK-Sym for symptomatic patients (such as those with chronic pancreatitis) who are at risk of developing PDAC.

**What do these findings mean?:**

- The urine biomarker panel and the affiliated PancRISK score were successfully validated in the large number of retrospectively collected samples and present a promising approach for completely non-invasive early detection of PDAC.
- After testing in a prospective cohort, this patient stratification approach could be implemented in clinical practice, with the potential to improve the current diagnostic pathway for PDAC patients.

## Introduction

Pancreatic ductal adenocarcinoma (PDAC) remains one of the most aggressive and incurable malignancies. With over 80% of cases diagnosed at advanced stages, PDAC patients have a median survival of 5–6 months, and 5-year survival rates around 9% have been reported globally [1–3]. However, if PDAC is detected earlier, when still localised, the 5-year survival rate can be greatly improved, up to 32% [3], approaching 70% following resection in incidentally diagnosed stage I tumours [4,5]. Currently, no useful biomarkers for earlier detection of PDAC exist; the only biomarker in clinical practice, serum CA19-9, is not specific or sensitive enough for screening purposes [6], and is mainly used as a prognostic marker and for monitoring response to treatment [7–9]. A large number of PDAC biomarkers have been investigated, alone or in combination with CA19-9; however, none seems to have reached prospective clinical testing [10–15].

While blood has traditionally been the main source of biomarkers, urine represents a promising alternative biological fluid [16,17]. It allows a completely non-invasive sampling, high volume collection, and ease of repeated measurements; it has a lesser dynamic range, with a less complex proteome than blood [18,19]. Furthermore, it is expected that the continuous ultrafiltration of blood by the kidneys would result in accumulation, and thus higher concentration, of at least some of the biomarkers in urine [16]. Despite this, urine remains relatively unexplored in the biomarker arena, due to the potential influence on biomarker levels of confounding factors such as impaired kidney function. While only a few biomarker discovery studies in PDAC urine samples are published to date, including ours [20–26], the urine proteome has also shown the potential to provide biomarkers for other non-urological cancers such as colon cancer [27], ovarian cancer [28], lung cancer [29], and cholangiocarcinoma [30].

In the present study, we validated our improved urinary panel not only on an increased number of cancer specimens, but also on samples obtained from symptomatic patients with chronic pancreatitis (CP) and other benign hepatobiliary diseases, which represent a challenge in early detection of PDAC because of the overlapping symptoms.

Furthermore, we tested how robust our biomarkers are by studying daily variations in their levels and their stability in urine. Finally, the obtained sensitivity (SN) and specificity (SP) data were used to select the most appropriate cutoffs for our recently developed algorithm, PancRISK [31], which was further tested in combination with CA19-9. PancRISK is a logistic regression model based on our 3 biomarkers, urine creatinine, and age, which enables stratification of patients into those with ‘normal’ or ‘elevated’ risk for developing PDAC.

## Methods

### Clinical specimens

#### Sample selection

In this case–control study [32], we utilised retrospectively collected urine and plasma samples for the validation of a urinary biomarker panel previously discovered using mass spectrometry [21]. Of the 590 urine specimens analysed, 183 were from control individuals (control group) who had no known pancreatic conditions or malignancies or history of renal diseases at the time of collection, 208 were from patients with benign hepatobiliary diseases (benign group), and 199 were from PDAC patients. All samples were collected before surgery or chemotherapeutic treatment and were age- and sex-matched wherever possible. benign samples included 119 CP cases, 54 gallbladder diseases, 20 cystic lesions of the pancreas, and 15 cases with abdominal pain and gastrointestinal symptoms suggestive of pancreatic origin (S1 Table). Of the 590 samples, 332 (81 control, 89 benign, and 162 PDAC) have been previously analysed [21]. The demographic details of the samples are described in Table 1. Centre of origin, details of diagnosis, and sample overlap are reported in S1 Table. Matched plasma specimens were available for 350 samples (92 control, 108 benign, and 150 PDAC) (see Tables 1 and S1 for details). A smaller subset of these (10 control, 10 benign, and 14 PDAC) was used to measure the levels of our urinary biomarkers in plasma.

**Table 1:** Demographics and clinical characteristics of patients and control individuals.

Sample type	Control			Benign			PDAC			
	Sample (*n*)	Sex (*n*)	Age range in years by sex (median)	Sample (*n*)	Sex (*n*)	Age range in years by sex (median)	Sample (*n*)	Sex (*n*)	Age range in years by sex (median)	Cancer stage (*n*)
Urine (total *n* = 590)	183	F = 115	26–89 (58)	208	F = 101	26–82 (53)	199	F = 83	42–88 (68)	I–IIA = 27; IIB = 75; III = 76; IV = 21
		M = 68	30–87 (55)		M = 107	29–82 (55)		M = 116	29–87 (67)	
		F + M	26–89 (57)		F + M	26–82 (54)		F + M	29–88 (67)	
Plasma (total *n* = 350)	92	F = 58	26–84 (60)	108	F = 57	26–77 (52)	150	F = 66	42–82 (68)	I–IIA = 20; IIB = 60; III = 65; IV = 5
		M = 34	30–87 (52.5)		M = 51	29–73 (54)		M = 66	29–83 (67)	
		F + M	26–87 (58)		F + M	26–77 (53)		F + M	29–83 (67)	

F, female; M, male; PDAC, pancreatic ductal adenocarcinoma.

In addition, 67 urine specimens included in the study were collected from patients with common urological tract malignancies: 18 from patients with prostate cancers (PC) (median age 69 years, range 52–83), 29 from patients with renal cell carcinoma (RCC) (median age 67 years, range 20–85), and 20 from patients with bladder transitional cell cancer (TCC) (median age 65 years, range 44–81). Finally, a total of 64 urine specimens from 4 control donors (3 females, 1 male) were used to establish the daily variation in the concentration of the biomarkers (*n* = 24).

#### Ethics statement

Clinical specimens were obtained from multiple centres after the respective institutional review board approvals: Barts Pancreas Tissue Bank (bartspancreastissuebank.org.uk; South Central Hampshire B Ethics Committee, ref number 13/SC/0592; project 2017/11/QM/TC/C/P approved by Tissue Access Committee); University College London (UCL); the Department of Molecular and Clinical Cancer Medicine, Liverpool University; and the Spanish National Cancer Research Center (Centro Nacional de Investigaciones Oncológicas [CNIO], Madrid, Spain). Samples from patients with bladder TCC were provided by the Institute of Medical and Clinical Biochemistry, Faculty of Medicine, University of Belgrade, and PC and RCC samples were obtained from the DIAMOND study, Cambridge University Hospitals NHS Trust (Ethics 03/18). All clinical specimens were obtained after individual written informed consent. The ethical approval for this study was obtained from London Brent Research Ethics Committee, reference number 05/Q0408/65.

#### Preservation and storage

Both collection and storage of urine and plasma samples were performed according to standard operating procedures, as previously described [21]. Clean-catch midstream urine was kept on ice upon collection, aliquoted before freezing within 2 hours from collection, and maintained at −80°C for long-term storage. For testing the potential effect of bacterial growth on the urine biomarkers, 20 control urine samples were collected as described above but with the addition of 20 mg/ml boric acid to the containers before collection. Blood samples were obtained at the same time as urine specimens. Blood was collected in EDTA vacutainer tubes and processed within 4 hours: after centrifugation at 1,500*g* for 10 minutes at room temperature, 0.5-ml aliquots of supernatant plasma were transferred into clean sterile 1.0-ml cryotubes and stored at −80°C. Specimens were shipped between centres on dry ice.

### Urine biomarkers and plasma CA19-9 measurements

All the assays have been performed following the protocol described below. An aliquot of frozen urine or plasma for each sample was thawed at 4°C before the experiments. Samples were processed in groups of 40 to fill 1 ELISA plate. Each measurement was performed in duplicate, and further repeats were run when there was a discrepancy between the duplicates. Commercially sourced ELISA kits were used for assaying the biomarkers: Cloud-Clone (Cat# SEB049HU) for TFF1 (1:100 and 1:300 urine dilutions), Sino Biological (Cat# ABIN2010491) for REG1B (1:500 and 1:1,000 urine dilutions), RayBiotech (Cat# ELH-LYVE1-001) for LYVE1 (1:10 and 1:20 urine dilutions), and RayBiotech (Cat# ELH-CA19-9-001) for CA19-9 (1:5 and 1:10 urine dilutions), all following the manufacturer’s instructions. TMB Substrate Set and Stop Solution from BioLegend (Cat# 421101 and 423001) were used for REG1B quantification. Plasma samples were diluted 1:5 and 1:10 for all 4 ELISAs. Optical density was determined using the FLUOstar Omega Microplate Reader. The limits of detection reported were 3.91, 8, and 56 pg/ml for TFF1, REG1B, and LYVE1, respectively. The minimum detectable level of CA19-9 was 0.3 U/ml. Intra- and inter-assay coefficients of variation were <10% and <12%, respectively, for all the assays. Plasma CA19-9 was also measured at The Doctors Laboratory, London, using the Roche platform (Cobas 601E [ECLIA] technology) according to routine protocols. Urine creatinine was measured at the Clinical Biochemistry Laboratory of the University of Westminster using an ILab Aries analyser from Instrumentation Laboratory according to the manufacturer’s protocol (limit of detection: 0.6 mmol/l). All the assays were performed by research staff who were blinded to the sample diagnosis.

### Statistical analysis

The biomarker values obtained by ELISAs were normalised and analysed as described previously [23]. Briefly, for multiple comparisons between the experimental groups, Kruskal–Wallis tests (with Dunn’s correction) were used. Spearman correlation was used to calculate the correlation between plasma and urine CA19-9. Both analyses were performed and graphs plotted using GraphPad Prism (version 8). The chi-squared test and analysis of variance were used to assess the difference in proportions of males and females and age across diagnosis groups.

The urine biomarker risk score PancRISK was previously developed based on a risk model with 5 predictors (LYVE1, REG1, TFF1, creatinine, and age) [29]. As in this study, we aimed to evaluate the performance of the model in a separate dataset and to compare it to CA19-9; data on 590 patient samples (183 control, 208 benign, and 199 PDAC) will allow us to achieve reliable estimates of the measures of discrimination. As 10 events per variable are needed for a powered study [33], at least 60 (5 predictors + CA19-9) samples for each group (control, benign hepatobiliary diseases, and PDAC) are required in order to estimate the SP and SN of the urine panel tested, which is satisfied in this study.

All protein concentration data were natural-log-transformed and mean-centred prior to the analysis. The biomarker panel was investigated for its ability to discriminate between PDAC at different stages and control and benign specimens using a receiver operating characteristics (ROC) curve analysis approach. Internal validation was performed by splitting the whole dataset into the training and validation sets in a 1:1 ratio. Logistic regression was applied to the training and the validation sets obtained after random division of the whole available dataset in a 1:1 ratio: one group comprising control and PDAC samples, the other comprising benign and PDAC samples (details and number of samples per group are provided in S2 Table). For both analyses, the model was fitted for the corresponding training set using the 5 predictors: 3 urinary biomarkers together with creatinine and age, creating a PancRISK score. Bootstrap cross-validation was used for the internal validation to ensure that overfitting was avoided. The performance characteristics of PancRISK were evaluated and compared in terms of the area under the ROC curve (AUC). The SP at a fixed SN was used in the analysis of control and PDAC samples, and SN at fixed SP for the analysis of benign and PDAC samples. The performance of the models used in this study was assessed at clinically relevant SN cutoffs for the analysis of asymptomatic patients and SP cutoffs for the analysis of symptomatic patients (both set at >80%). Confidence intervals (95% CIs) for AUCs were derived based on DeLong’s asymptotically exact method to evaluate the uncertainty of an AUC [34]; SN and SP and 95% CI were derived using non-parametric stratified resampling with the percentile method (2,000 bootstrap replicates) as described by Carpenter and Bithell [35]. AUCs were compared using the DeLong’s 1-sided test for correlated/paired AUCs [34].

Positive and negative predictive values (PPV and NPV) for a number of prevalence estimates were calculated using the standard approach [36].

In a subset of the data, where both urine and plasma were available, we compared the PancRISK score to CA19-9 performance and a combination of both. Logistic regression was applied to the panel, to CA19-9, and to their combination. Performance characteristics included AUC as well as SP at fixed SN and SN at fixed SP for the comparisons of control and benign samples with PDAC samples, respectively. In addition to the conventional 37-U/ml cutoff for CA19-9, we also investigated the potential complementarity of PancRISK and CA19-9 at other possible cutoffs.

All analyses were performed in R version 3.5.1 (R Foundation for Statistical Computing; http://www.r-project.org/foundation/) using the ROCR [37] and pROC packages [38].

The flow diagram of this study and the REMARK checklist document are provided as S1 Appendix and S2 Appendix, respectively.

The analysis was performed according to the prespecified plan summarised in the flow diagram reported in S1 Appendix. No data-driven changes took place after the analysis started.

## Results

### Urine REG1B outperforms REG1A in detecting early stage PDAC

In our previous study [23], proteomic data identified both REG1A and REG1B as potential urinary biomarker candidates, with the latter showing better differential. However, the previous panel included REG1A, as commercially available REG1B ELISA only became available towards the end of the study. To more firmly establish which of these 2 biomarkers has a superior performance, a more detailed comparison of the 2 proteins was performed using a subset of 306 samples. REG1A values used for the comparison were taken from Radon et al. [23]. The detailed information on the samples and the data are reported in S1 Table. It is evident that while their performance was similar, REG1B outperformed REG1A in the comparison between control samples and stage I–IIA PDAC samples (*p* = 0.032, Kruskal–Wallis test) (S1 Fig). Therefore, all experiments subsequent were performed using REG1B as a constitutive part of the biomarker panel.

### Urine biomarker panel performance in detecting PDAC

The panel was tested in 590 retrospectively collected urine specimens (183 control, 208 benign, and 199 PDAC). It is evident from Table 1 that the differences in proportions of males and females and age across the experimental groups were statistically significant (*p* = 0.015 for difference in sex across diagnosis groups in plasma and *p* < 0.001 for the remaining comparisons); however, as our urinary biomarkers were selected as being differentially expressed in the experimental groups in both sexes [21], and age is included as an additional predictor in all the models, these differences in demographics do not impact our results.

**Fig 1:**
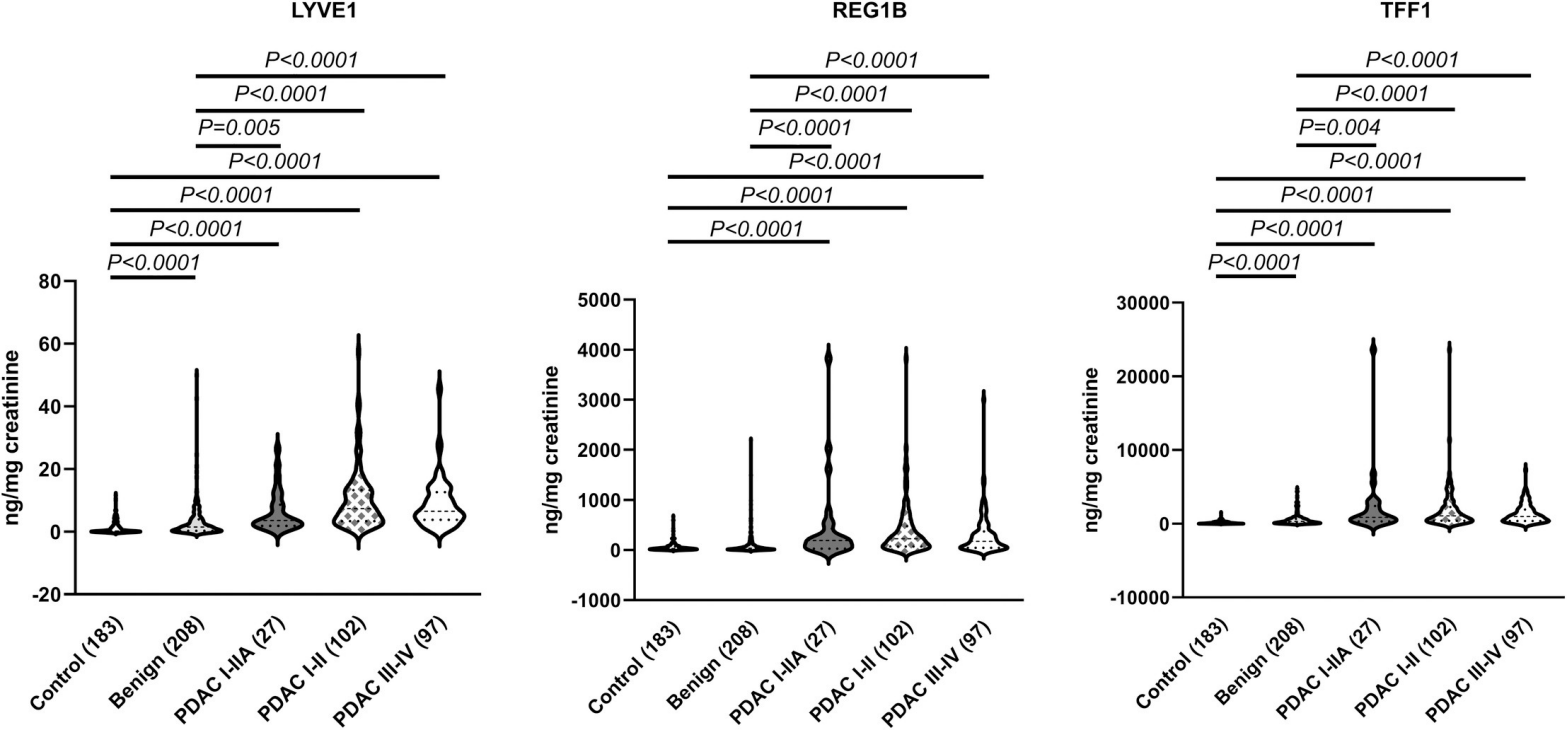
The levels of the 3 biomarkers in control, benign, and pancreatic ductal adenocarcinoma (PDAC) samples. Violin plots are shown for each protein. The number of samples per group is shown in parentheses. All data were creatinine normalised. Upper bars: Kruskal–Wallis test, Dunn’s multiple comparisons.

While some of the samples overlap with our previous report [23], due to changes in the TFF1 and LYVE1 commercially sourced ELISAs (in both cases different quantification ranges in changed kit versions were seen), all the samples had to be re-assayed. Despite these differences, the analyses similarly confirmed a significantly higher concentration of all 3 biomarkers in PDAC urine specimens at all stages (102 I–II and 97 III–IV), when compared with both benign and control samples (Kruskal–Wallis test, *p* < 0.0001) (Fig 1). While the biomarker values were higher in earlier PDAC stages, this difference did not reach statistical significance. A complete list of all raw data obtained from ELISAs for the 3 biomarkers is provided in S1 Table.

### Performance of the panel in differentiating control individuals and those with benign conditions from PDAC patients

**Fig 2:**
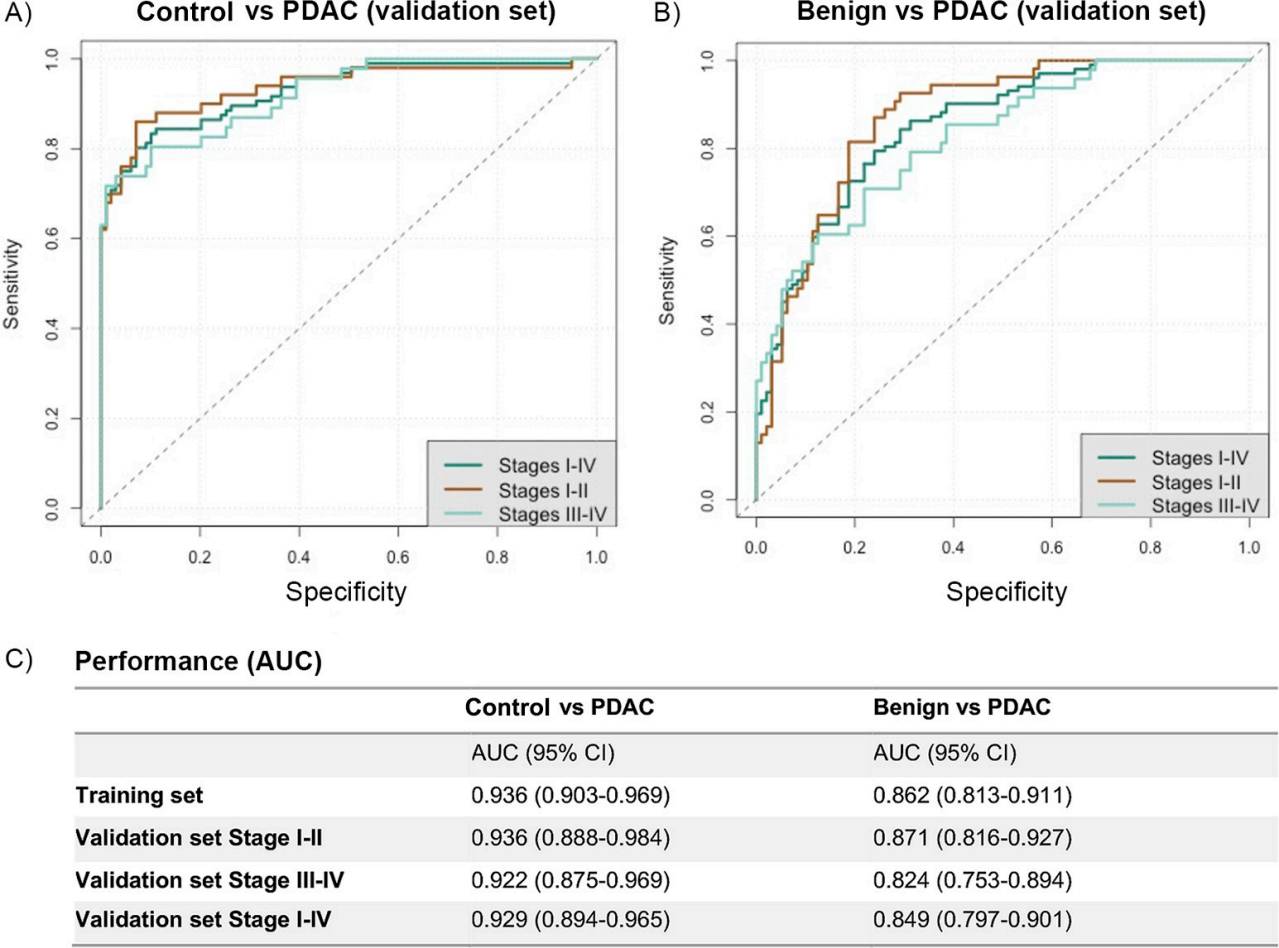
Performance of urine biomarker panel. Comparison of pancreatic ductal adenocarcinoma (PDAC) stages I–II, III–IV, and I–IV to control samples (A and C) and to benign samples (B and C). Receiver operating characteristic (ROC) curves of the 3-biomarker panel in the validation set (50% of the samples) for PDAC I–II, III–IV, and I–IV in the comparison with control (A) and benign (B) samples. Performance of the biomarkers in the training and validation sets is expressed as area under the ROC curve (AUC) (C).

Using a ROC curve analysis, the performances of the 3-biomarker panel in discriminating between PDAC stages I–II, III–IV, and I–IV and control urine were established in a training dataset (50% of the samples, comprising 84 control, 52 PDAC I–II, and 51 PDAC III–IV) (Fig 2A; S2 Table). The panel, adjusted for age and creatinine, resulted in an AUC of 0.936 (95% CI 0.903–0.969) in the training set and in AUCs of 0.936 (95% CI 0.888–0.984), 0.922 (95% CI 0.875–0.969), and 0.929 (95% CI 0.894–0.965) in the validation dataset (50% of the samples, comprising 99 control, 50 PDAC I–II, and 46 PDAC III–IV) in comparisons between control samples and PDAC I–II, III–IV, and I–IV stage samples, respectively (Fig 2A and 2C; S2 Table).

The performance of the panel in discriminating patients with PDAC stages I–II, III–IV, and I–IV from patients with benign hepatobiliary diseases in a training dataset (50% of the samples, comprising 112 benign, 48 PDAC I–II, and 49 PDAC III–IV) is shown on Fig 2B: an AUC of 0.862 (95% CI 0.813–0.911) in the training set and AUCs of 0.871 (95% CI 0.816–0.927), 0.824 (95% CI 0.753–0.894), and 0.849 (95% CI 0.797–0.901) in the validation dataset (50% of the samples, comprising 96 benign, 54 PDAC I–II, and 48 PDAC III–IV) were obtained when benign samples were compared to PDAC stages I–II, III–IV, and I–IV, respectively (Fig 2C).

In order to select the most appropriate cutoffs for the PancRISK score, we explored the dynamic changes in SN and SP in the validation set after presetting their values (Tables 2 and 3). The resulting SP at fixed SN in the comparison of control samples with PDAC I–II samples and SN at fixed SP in the comparison of benign samples with PDAC I–II samples are reported in Tables 2 and 3, respectively.

**Table 2:** Performance of the urinary panel with different cutoffs: Specificity at fixed sensitivity in the validation set.

Sensitivity cutoff	Specificity		
	Control versus PDAC I–II	Control versus PDAC III–IV	Control versus PDAC I–IV
0.80	0.939 (0.808–1.000)	0.889 (0.626–1.000)	0.919 (0.748–0.990)
0.85	0.919 (0.677–0.980)	0.727 (0.556–0.950)	0.828 (0.657–0.960)
0.90	0.818 (0.596–0.970)	0.646 (0.505–0.879)	0.707 (0.556–0.909)
0.95	0.636 (0.040–0.909)	0.596 (0.424–0.748)	0.586 (0.414–0.727)

PDAC, pancreatic ductal adenocarcinoma.

**Table 3:** Performance of the urinary panel with different cutoffs: Sensitivity at fixed specificity in the validation set.

Specificity cutoff	Sensitivity		
	Benign versus PDAC I–II	Benign versus PDAC III–IV	Benign versus PDAC I–IV
0.80	0.796 (0.574–0.944)	0.646 (0.479–0.813)	0.725 (0.569–0.853)
0.85	0.667 (0.426–0.889)	0.604 (0.438–0.750)	0.637 (0.480–0.794)
0.90	0.519 (0.315–0.759)	0.542 (0.375–0.708)	0.529 (0.382–0.706)
0.95	0.370 (0.093–0.574)	0.417 (0.250–0.626)	0.382 (0.196–0.579)

PDAC, pancreatic ductal adenocarcinoma.

Because the precise prevalence of PDAC is not known, positive and negative predictive values (PPV and NPV) for a number of prevalence estimates were calculated in the validation set for control samples and benign samples versus PDAC samples (S3 Table). A high negative predictive value, excluding the majority of patients with benign conditions that will have a ‘normal’ risk for pancreatic cancer, is demonstrated.

### Performance of the biomarkers in combination with CA19-9

**Fig 3:**
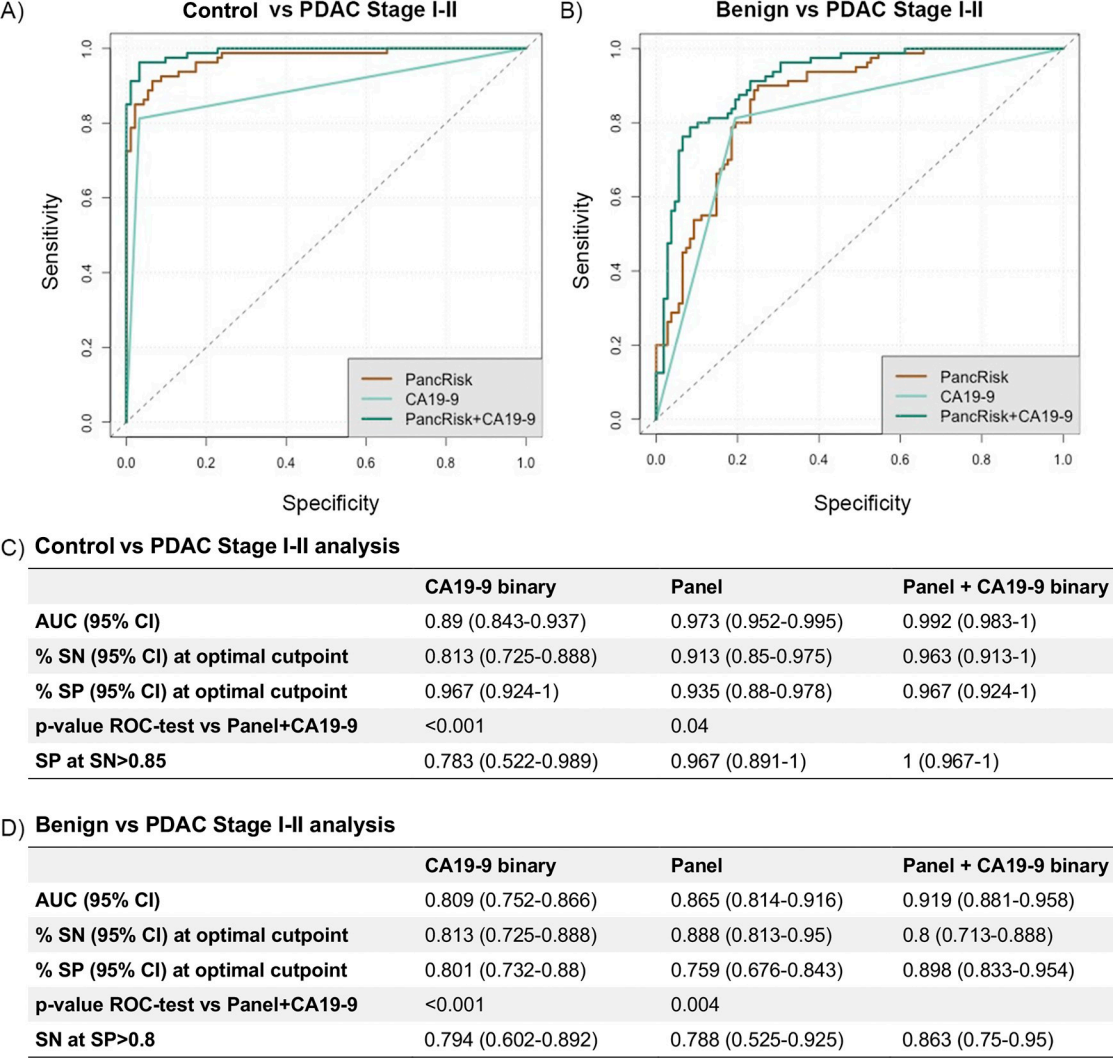
Evaluation of the urinary panel in combination with plasma CA19-9 for PDAC I–II discrimination. Performance in distinguishing early stage PDAC (stage I–II) (*n* = 80) from control samples (*n* = 92) (A and C) and benign samples (*n* = 108) (B and D). ROC curves of CA19-9 and biomarker panel alone and in combination (A and B); summary of the performances (C and D). AUC, area under the receiver operating characteristic curve; CI, confidence interval; PDAC, pancreatic ductal adenocarcinoma; ROC, receiver operating characteristic; SN, sensitivity; SP, specificity.

Plasma CA19-9 levels were available for 92 control, 108 benign, 80 PDAC I–II, and 70 PDAC III–IV samples. Using this subset of samples, we first evaluated the performance of the panel with and without CA19-9 in discriminating between control and PDAC stage I–II samples (Fig 3A). The AUC for CA19-9 (at a 37-U/ml cutoff) in combination with the panel was 0.992 (95% CI 0.983–1.000) (SN = 0.963, 95% CI 0.913–1.000; SP = 0.967, 95% CI 0.924–1.000), which was significantly greater than the AUCs obtained for the urine panel and plasma CA19-9 alone (*p* = 0.04 and *p* < 0.001, respectively) (Fig 3C).

In the comparison of benign versus PDAC I–II samples, the AUC for the combination of plasma CA19-9 and the panel increased significantly to 0.919 (95% CI 0.881–0.958) (SN = 0.80, 95% CI 0.713–0.888, and SP = 0.898, 95% CI 0.833–0.954) when compared to plasma CA19-9 and the urinary panel alone (*p* < 0.001 and *p* = 0.004, respectively) (Fig 3B and 3D).

Improved performance was also observed when the urinary panel was analysed in combination with plasma CA19-9 in discriminating between control samples and both late stage PDAC (stage III–IV; S2A and S2C Fig) and all stage PDAC (S2B and S2D Fig). In the latter, AUCs for CA19-9 alone and for the panel significantly increased when in combination (*p* = 0.002 and *p* < 0.001, respectively) (S2D Fig). CA-19-9 also increased the performance of the panel in discriminating benign versus stages III–IV and I–IV (S3 Fig).

**Fig 4:**
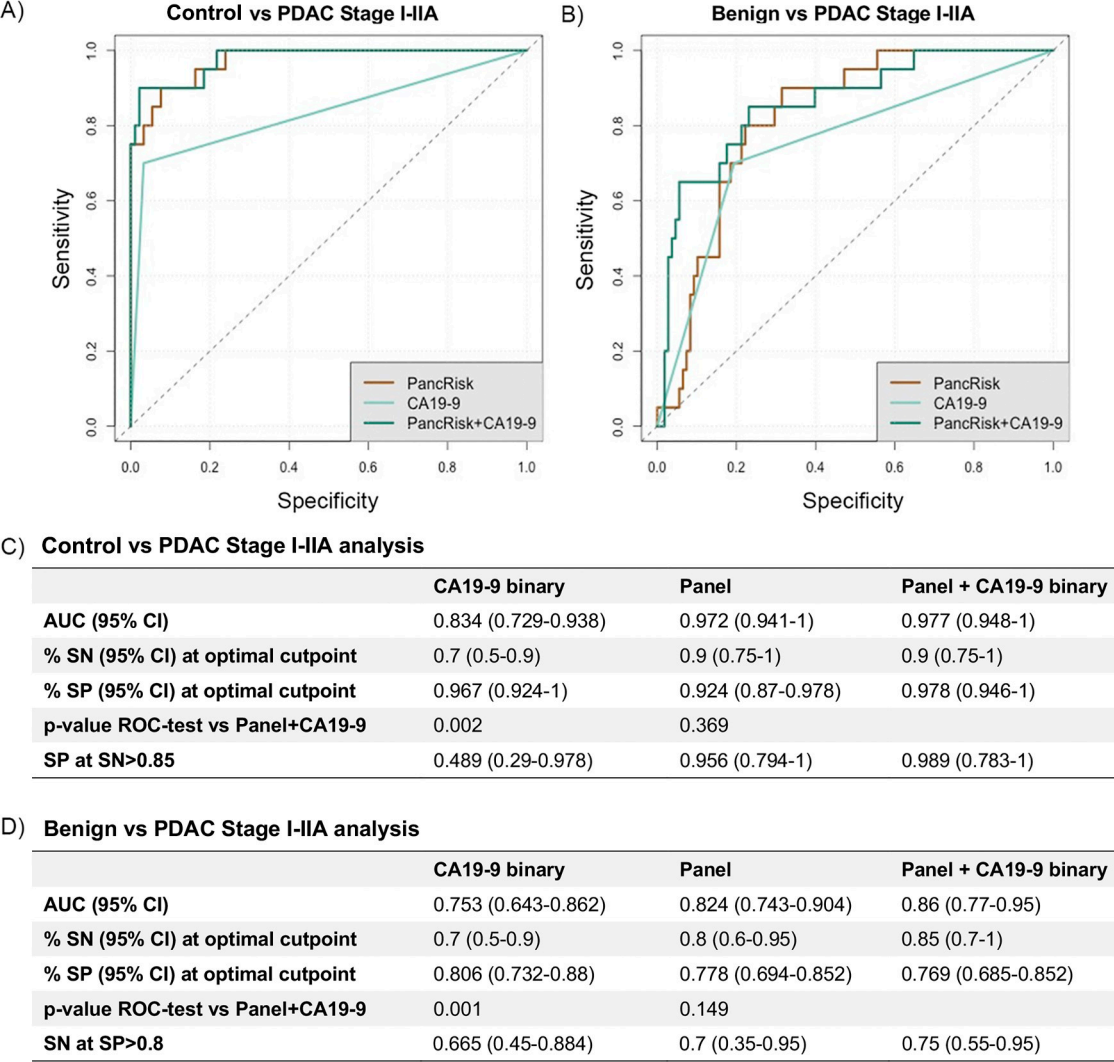
Evaluation of the urinary panel in combination with plasma CA19-9 for PDAC I–IIA discrimination. Performance in distinguishing PDAC stage I–IIA (*n* = 20) from control samples (*n* = 92) (A and C) and benign samples (*n* = 108) (B and D). ROC curve of CA19-9 and biomarker panel alone and in combination (A and B); summary of the performances (C and D). AUC, area under the receiver operating characteristic curve; CI, confidence interval; PDAC, pancreatic ductal adenocarcinoma; ROC, receiver operating characteristic; SN, sensitivity; SP, specificity.

Due to limited number of stage I–IIA PDAC samples, where the tumour has not yet spread to the lymph nodes (*n* = 27 for urine, *n* = 20 for matched urine and plasma), we could only perform an exploratory analysis, but the addition of CA19-9 did not significantly improve the panel in distinguishing cancer from control samples (AUC = 0.977, 95% CI 0.948–1.000) (Fig 4A and 4C) or benign samples (AUC = 0.860, 95% CI 0.770–0.950) (*p* = 0.369 and *p* = 0.149, respectively) (Fig 4B and 4D).

### Subset analysis of the performance of the panel in differentiating PDAC from CP patients

The performance of the panel in discriminating patients with PDAC from 119 patients with CP, a subset of the benign hepatobiliary disease samples, was calculated for the training and validating datasets (S4 Fig). The panel, adjusted for age and creatinine, resulted in an AUC of 0.873 (95% CI 0.818–0.929) in the training set (50% of the samples) (S4A Fig) and in AUCs of 0.818 (95% CI 0.740–0.896), 0.808 (95% CI 0.725–0.891), and 0.814 (95% CI 0.744–0.883) in the validation dataset (50% of the samples) in the comparisons between CP and PDAC I–II, III–IV, and I–IV stages, respectively (S4B Fig). SNs, calculated at fixed SP cutoffs in the validation set, were slightly lower than those for discriminating benign samples from PDAC samples.

The performance of the panel with or without CA19-9 in discriminating PDAC stages I–II, III–IV, and I–IV in 64 CP samples with data available for plasma CA19-9 was also calculated; the results are reported in S5 Fig. As already noted for control and benign samples, CA19-9 enhanced the performance of the panel in the discrimination of CP from each PDAC stage group, with AUCs very close to those achieved using the whole benign group of samples (S5D–S5F Fig).

### Performance of PancRISK in combination with different CA19-9 cutoffs

Based on obtained data (Tables 2 and 3), we have constructed 2 versions of PancRISK. The first one, PancRISK-Fam, is aimed at stratifying the asymptomatic patients at risk (familial history, genetic syndromes), for which we have therefore set the SN cutoff at 0.85 in order to detect the cases. For the second one, called PancRISK-Sym, we have preset the SP cutoff at 0.80, as it is aimed at stratification of symptomatic patients. The patients with ‘normal’ risk should not undergo further invasive workup, while the ones with ‘elevated’ risk should continue to be investigated. Tables 4 and 5 show the performance of PancRISK-Fam and PancRISK-Sym, respectively, when combined with CA19-9 at different cutoffs (37 U/ml, 40 U/ml, 45 U/ml, and 60 U/ml). The tables demonstrate that increasing the CA19-9 cutoff led to a small decrease in SN but increase in SP of PDAC detection.

**Table 4:** Performance of the PancRISK in combination with different CA19-9 cutoffs: PancRISK-Fam (sensitivity = 0.85).

Population and PancRISK group	Lower CA19-9 cutoff group and *n*	Upper CA19-9 cutoff group and *n*	Specificity and sensitivity
**Control (*n* = 92)**	CA19-9 < 37 U/ml	CA19-9 ≥ 37 U/ml	Specificity: 0.891 (0.811–0.940)
PancRISK = ‘normal’	82	3	
PancRISK = ‘elevated’	7	0	
**PDAC (*n* = 150)**	CA19-9 < 37 U/ml	CA19-9 ≥ 37 U/ml	Sensitivity: 0.967 (0.924–0.986)
PancRISK = ‘normal’	5	17	
PancRISK = ‘elevated’	16	112	
**Control (*n* = 92)**	CA19-9 < 40 U/ml	CA19-9 ≥ 40 U/ml	Specificity: 0.902 (0.824–0.948)
PancRISK = ‘normal’	83	2	
PancRISK = ‘elevated’	7	0	
**PDAC (*n* = 150)**	CA19-9 < 40 U/ml	CA19-9 ≥ 40 U/ml	Sensitivity: 0.960 (0.915–0.982)
PancRISK = ‘normal’	6	16	
PancRISK = ‘elevated’	16	112	
**Control (*n* = 92)**	CA19-9 < 45 U/ml	CA19-9 ≥ 45 U/ml	Specificity: 0.902 (0.824–0.948)
PancRISK = ‘normal’	83	2	
PancRISK = ‘elevated’	7	0	
**PDAC (*n* = 150)**	CA19-9 < 45 U/ml	CA19-9 ≥ 45 U/ml	Sensitivity: 0.960 (0.915–0.982)
PancRISK = ‘normal’	6	16	
PancRISK = ‘elevated’	19	109	
**Control (*n* = 92)**	CA19-9 < 60 U/ml	CA19-9 ≥ 60 U/ml	Specificity: 0.913 (0.838–0.955)
PancRISK = ‘normal’	84	1	
PancRISK = ‘elevated’	7	0	
**PDAC (*n* = 150)**	CA19-9 < 60 U/ml	CA19-9 ≥ 60 U/ml	Sensitivity: 0.960 (0.915–0.982)
PancRISK = ‘normal’	6	16	
PancRISK = ‘elevated’	23	105	

PDAC, pancreatic ductal adenocarcinoma.

**Table 5:** Performance of the PancRISK in combination with different CA19-9 cutoffs: PancRISK-Sym (specificity = 0.80).

Population and PancRISK group	Lower CA19-9 cutoff group and *n*	Upper CA19-9 cutoff group and *n*	Specificity and sensitivity
**Benign (*n* = 108)**	CA19-9 < 37 U/ml	CA19-9 ≥ 37 U/ml	Specificity: 0.676 (0.583–0.757)
PancRISK = ‘normal’	73	14	
PancRISK = ‘elevated’	14	7	
**PDAC (*n* = 150)**	CA19-9 < 37 U/ml	CA19-9 ≥ 37 U/ml	Sensitivity: 0.947 (0.898–0.973)
PancRISK = ‘normal’	8	41	
PancRISK = ‘elevated’	13	88	
**Benign (*n* = 108)**	CA19-9 < 40 U/ml	CA19-9 ≥ 40 U/ml	Specificity: 0.685 (0.593–0.765)
PancRISK = ‘normal’	74	13	
PancRISK = ‘elevated’	14	7	
**PDAC (*n* = 150)**	CA19-9 < 40 U/ml	CA19-9 ≥ 40 U/ml	Sensitivity: 0.947 (0.898–0.973)
PancRISK = ‘normal’	8	41	
PancRISK = ‘elevated’	14	87	
**Benign (*n* = 108)**	CA19-9 < 45 U/ml	CA19-9 ≥ 45 U/ml	Specificity: 0.704 (0.612–0.782)
PancRISK = ‘normal’	76	11	
PancRISK = ‘elevated’	14	7	
**PDAC (*n* = 150)**	CA19-9 < 45 U/ml	CA19-9 ≥ 45 U/ml	Sensitivity: 0.940 (0.890–0.968)
PancRISK = ‘normal’	9	40	
PancRISK = ‘elevated’	16	85	
**Benign (*n* = 108)**	CA19-9 < 60 U/ml	CA19-9 ≥ 60 U/ml	Specificity: 0.741 (0.651–0.814)
PancRISK = ‘normal’	80	7	
PancRISK = ‘elevated’	14	7	
**PDAC (*n* = 150)**	CA19-9 < 60 U/ml	CA19-9 ≥ 60 U/ml	Sensitivity: 0.940 (0.890–0.968)
PancRISK = ‘normal’	9	40	
PancRISK = ‘elevated’	20	81	

PDAC, pancreatic ductal adenocarcinoma.

### Expression of CA19-9 and 3 biomarkers in urine and plasma

Since CA19-9 can improve the performance of our biomarkers (except for stages I–IIA), we next wanted to establish the possibility of detecting CA19-9 in urine. We measured CA19-9 in matched plasma and urine samples by both Roche Cobas instrument and RayBiotech ELISAs in a subset of 78 samples (22 control, 9 benign, and 47 PDAC) (S4 Table). Only plasma CA19-9 was able to distinguish PDAC samples from both control samples (*p* < 0.0001) and benign samples (*p* = 0.002) (S6A Fig), whereas, despite the visible pattern, none of the differences between these experimental groups reached statistical significance in urine with either the Roche Cobas method (S6B Fig) or ELISA (S6C Fig). There was no correlation between plasma and urine CA19-9 measured by Roche or ELISA (Spearman correlation of 0.525, CI 95% 0.337–0.673, *p* < 0.0001, and 0.367, CI 95% 0.151–0.550, *p* < 0.001, respectively).

Similarly, when a smaller subset of the matched plasma samples (10 control, 10 benign, 14 PDAC) was used to measure the levels of our urinary biomarkers, we observed a lower performance of our biomarkers in plasma than in urine (S7 Fig).

### The 3 protein biomarkers are stable in urine

With the aim of developing a robust biomarker panel for future clinical use, it is important to test the daily variation in the concentration and the stability of the 3 proteins in urine. For this, the biomarker levels were measured by ELISA in urine samples from 4 control donors, which were collected twice a day, in the morning before breakfast (AM) and in the afternoon after lunch (PM) over a 4-day period and immediately frozen after collection (S5 Table). No significant differences in any of the 3 proteins between collection times and over the 4 days could be seen (Kruskal–Wallis test) (S8A Fig).

Furthermore, the stability of the 3 biomarkers was assessed after keeping the urine samples at room temperature up to 5 days after collection in tubes with or without the addition of 20 mg/ml boric acid to supress bacterial growth (S5 Table). The concentration of all 3 biomarkers displayed no statistically significant variation (Kruskal–Wallis test) over the 5-day period in both types of tubes (S8B and S8C Fig) even when the Combur dipstick test (Diagnostics Roche) indicated bacterial growth after 3 days in urine collected in containers without boric acid.

### Biomarker expression in other cancers

**Fig 5:**
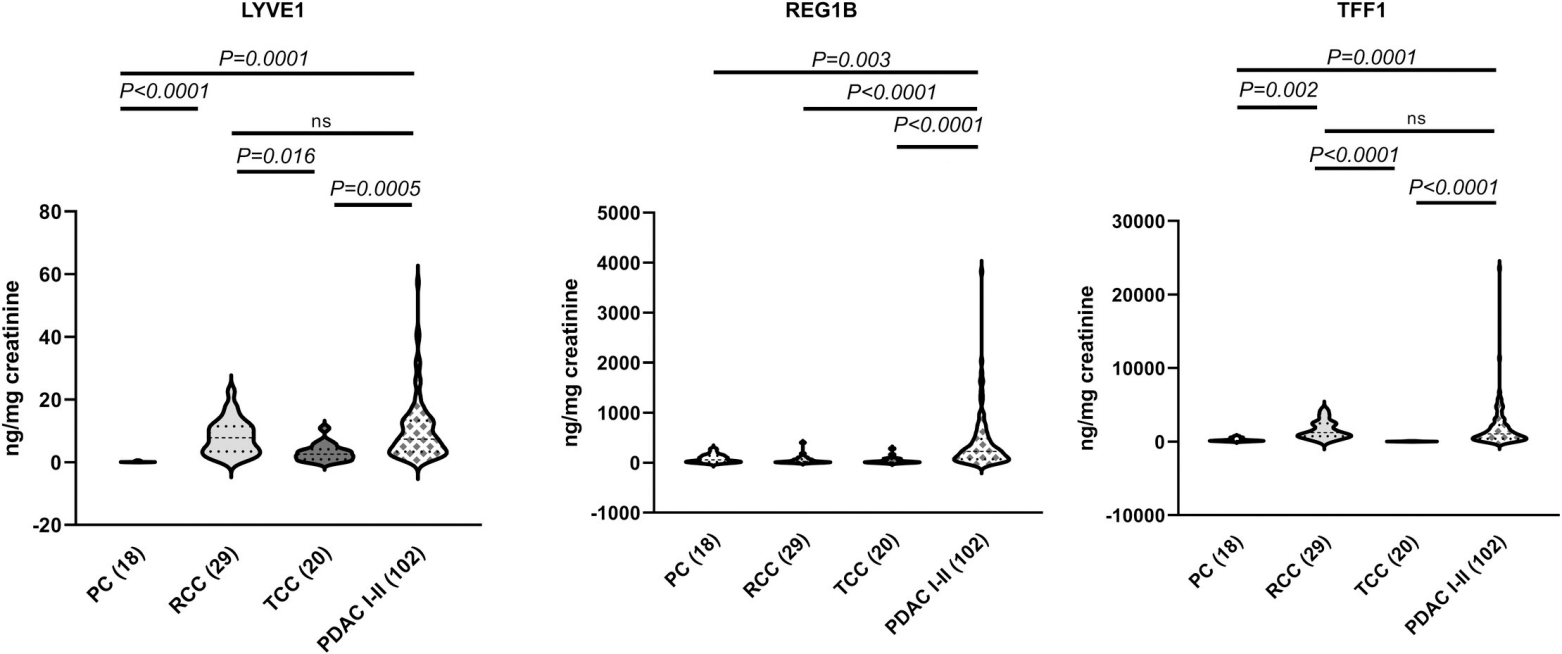
Biomarkers in common genitourinary cancers. The violin plots show the levels of the 3 urinary biomarkers in prostate cancer (PC), renal cell carcinoma (RCC), and bladder transitional cell carcinoma (TCC) compared with pancreatic ductal adenocarcinoma (PDAC) stages I–II. All data were creatinine normalised. The number of samples for each group is shown in parentheses. Upper bars: Kruskal–Wallis test, Dunn’s multiple comparisons; ns, not significant.

Finally, we tested the expression of the 3 biomarkers in common urinary tract cancers: 18 PC, 29 RCC, and 20 bladder TCC. The full set of data for each individual biomarker is shown in S6 Table. We compared the expression of the 3 biomarkers in other cancers with that in stage I–II PDAC (Fig 5).

We found that none of the biomarkers are significantly elevated in PC and in bladder TCC, while in RCC, LYVE1 and TFF1 levels were not significantly different to those in PDAC. This was due to the very high values of both LYVE1 and TFF1 in 3 patients, while an additional 2 patients demonstrated high values of either LYVE1 or TFF1 (S6 Table). These data indicate the overall good SP of our biomarkers for PDAC (Fig 5).

## Discussion

In this study we report on the performance of our enhanced urinary biomarker panel comprising LYVE1, REG1B, and TFF1 in a larger cohort of specimens, demonstrating its robust performance in discriminating patients with early stages of PDAC from control individuals and patients with benign hepatobiliary diseases. We also show that the performance of the panel was improved when combined with plasma CA19-9, and that the biomarkers do not show significant daily variation and are stable in urine.

LYVE1 (lymphatic vessel endothelial hyaluronan receptor 1) is a receptor that binds to both soluble and immobilised hyaluronan. It is involved in lymphatic hyaluronan transport [39] and has an active role in lymphangiogenesis and endothelial remodelling [40]. REG1B (regenerating family member 1 beta) belongs to a family of REG (regenerating) glycoproteins, a group of calcium-dependent proteins expressed in pancreatic acinar cells. They act as both autocrine and paracrine growth factors and have been described in patients with pancreatitis and during pancreatic islet regeneration [41,42]. TFF1 (trefoil factor 1) belongs to a family of gastrointestinal secretory peptides that interact with mucins and are expressed at increased levels during reconstitution and repair of mucosal injury. They protect epithelial cells from apoptotic death and increase their motility, but also play similar pivotal roles in cancer cells, and are thus involved in the development and progression of various cancer types [43,44].

All 3 biomarkers have been reported as upregulated in PDAC precursor lesions, i.e., pancreatic intraepithelial neoplasias (PanINs), and involved in cancer progression and metastasis. Using a transgenic model of PanINs, Shen et al. recently demonstrated the association of early lesions and lymphatic vessels with marked lymphangiogenesis and endothelial remodelling [40], and we have previously shown, in familial pancreatic cancer (FPC), that the overexpression of TFF1 was already present in early PanINs before cancer developed [45]. Both REG1A and REG1B upregulation was demonstrated in precursor lesions, which resulted in an accelerated cell proliferation and tumour growth [46].

While in our previous study [23] the panel included REG1A, we have now modified it by replacing REG1A with REG1B, as the latter protein outperforms REG1A in discriminating control samples from PDAC stage I–IIA samples. Of note, both proteins have previously been proposed as candidate biomarkers for pancreatic cancer [46–48]. Li et al. [46] showed that they are absent in normal tissue but are highly expressed in PDAC. While REG1A expression increases with progression from PanINs to cancer, REG1B is already highly expressed in the earliest PanINs, which is in agreement with our data showing that REG1B is an earlier marker. The same authors also reported a decreased tissue expression of the 2 proteins in late stage PDAC, and that the serum levels of both REG1A and REG1B were significantly higher in PDAC patients than in control individuals, but not in CP patients [46]. In contrast, our data show that urinary REG1B levels in PDAC are significantly higher than in both control individuals and patients with benign diseases (including CP).

Recently, Klett et al. [49] designed a pancreatic cancer classifier based on transcriptome analysis. Among the 17 genes, TFF1 was selected for its ability to distinguish normal pancreas from PanIN and PDAC tissues. The latter discrimination was validated in plasma (although only 40 samples were interrogated), thus supporting our finding that TFF1 is a good biomarker for PDAC detection.

The combination of plasma or serum CA19-9 with other biomarkers has been extensively explored [26,47,48,50]. Recently, Capello et al. [50] analysed CA19-9 with different combinations of 17 plasma proteins, while Mayerle et al. [26] combined CA19-9 with 9 different metabolites. Lee et al. [51] reported an improved CA19-9 performance when combined with CEMIP (cell migration inducing hyaluronidase). In the present study, as we showed previously [31], we found that plasma CA19-9 enhanced the performance of the panel in discriminating control samples and benign samples from stage I–II and late stage PDAC samples; however, this was not the case for the PDAC I–IIA samples, indicating superior performance of our urine panel for earlier detection of pancreatic cancer. Mellby et al. [12] drew a similar conclusion, based on a serum biomarker panel comprising 29 proteins, which showed the ability to discriminate early stage PDAC from control samples without inclusion of CA19-9.

Most of the biomarker studies published in PDAC are based on serum or plasma analyses. Only recently, a few publications have emerged highlighting the potential of urine in biomarker studies in the pancreatic field as well [52–55]. Roy et al. showed that urine MMP2 (matrix metallopeptidase 2) and TIMP-1 (tissue inhibitor of metalloproteinase 1) combined can differentiate PDAC (*n* = 50) from control samples (*n* = 60), with 91% SN and 75% SP [52], and Hogendorf et al. [53] showed that neutrophil gelatinase-associated lipocalin (NGAL), measured in urine can distinguish PDAC (*n* = 21) from CP (*n* = 15), with both SN and SP of 80%. Urinary PGEM (prostaglandin E2 metabolite) was recently proposed as a biomarker of PDAC risk prediction [54]. Yip-Schneider et al. [55] compiled a urinary panel with 3 of the previously reported biomarkers: LYVE1, TIMP-1, and PGEM. They tested the ability of these biomarkers to predict the progression of high-grade intraductal papillary mucinous neoplasm (IPMN) to cancer: While no statistically significant differences in expression levels for any of the 3 proteins were seen between low- and high-grade IPMN, in line with our findings, LYVE1 showed a significant difference in distinguishing control samples from PDAC samples.

In the present report we also show that our biomarkers are stable in urine, which is in line with previous proteomic studies, as reviewed by Kalantari et al. [56] and Tantipaiboonwong et al. [29]. While adding boric acid to the collection tubes can be beneficial in sample transport and for long-term storage, our results indicate that even with bacterial contamination our biomarkers are not affected. Such information is important for the development of a robust assay with the aim of future translation into the clinical setting.

Our aim was to develop a test for earlier non-invasive detection of PDAC using urine samples. We therefore tested if CA19-9 can also be detected in urine. However, although a pattern of increased expression of CA19-9 was seen in urine from PDAC patients, none of the differences among the experimental groups (control, benign diseases, and PDAC) reached statistical significance. Results similar to ours were also observed by another group, although they assayed only 5 urine samples [57]. Interestingly, urinary CA19-9 was found to be superior to its serum counterpart in the diagnosis of low-grade and early stages of TCC of urinary bladder [58]. Increased levels of CA19-9 were seen in urine due to urothelial obstruction [59] and renal injury [60], indicating the local production and secretion of this glycolipid into urine. Our inability to detect significant differences in CA19-9 expression in our experiments using pancreatic samples could therefore be due to either limited passage of this molecule through the glomerular barrier or post-translational modification or proteolytic processing that lead to masking, alteration, or loss of the epitopes recognised by currently available antibodies.

In order to achieve truly non-invasive PDAC detection, we are now testing additional analytes in urine to potentially combine with our protein panel [25,61].

One of the challenges in early detection of PDAC is the selection of the appropriate cohorts at risk, as screening in the general population is not feasible [62,63]. Current guidelines recommend screening of asymptomatic individuals with a history of FPC and predisposing genetic syndromes [64]. Additional amenable cohorts include patients with CP, cystic lesions of the pancreas, and new onset diabetes (NOD) [62]. We propose to test our biomarkers and PancRISK in all the above cohorts. For individuals with genetic predisposition, we would apply the PancRISK-Fam threshold based on SN, to maximise the chances of detecting PDAC, while in patients with symptoms suggestive of PDAC (such as CP), we would fix the threshold in PancRISK-Sym based on SP. The patients deemed by PancRISK to have an elevated risk of PDAC development would then undergo further, typically invasive and costly clinical workup, e.g., imaging. The simple urine testing with a binary PancRISK outcome would be straightforward to employ and would likely serve as a cost-effective triaging (sieving) tool, further enriching the populations at risk.

Despite the promising results, our study has several limitations. We interrogated a small number of urine samples collected from patients with PDAC I–IIA (*n* = 27), which was unfortunately unavoidable, since the large majority of patients are still diagnosed with advanced cancer. Also, we only tested our panel in a limited number of benign cystic lesions of the pancreas and have not performed the direct comparison to their invasive counterparts, again due to the unavailability of such samples. Similarly, our panel needs to be tested in urine collected from FPC and NOD cohorts as soon as they become available. A further drawback is the use of commercially available ELISAs, an issue which we already faced previously [31] and experienced again. Due to changes in ELISAs, we had to repeat all the measurements for both TFF1 and LYVE1. Since starting with the biomarker studies more than a decade ago, we have observed significant variability in ELISA performance for all 3 proteins, not only between different companies but also within the same manufacturer due to changes in the reagents and the capture and detection antibodies.

### Conclusions

In summary, we successfully validated the performance of our urinary biomarker panel in detecting PDAC earlier. Our PancRISK score is designed to enable further stratification of patients already predisposed to develop PDAC, thus enriching the population that should undergo follow-up, i.e., invasive clinical workup. We are now further evaluating the biomarkers and affiliated PancRISK score in a large prospective observational study, UroPanc (http://www.pcrf.org.uk/pages/uropanc-clinical-study.html).

## Supporting information

S1 Fig Comparison of REG1A and REG1B performance. Violin plots showing the levels of REG1A and REG1B in 306 urine samples (79 control, 87 benign, and 140 PDAC [16 stage I–IIA, 70 stage I–II, and 70 stage III–IV]). All data were creatinine normalised. Upper bars: Kruskal–Wallis test, Dunn’s multiple comparisons; ns, not significant.
(TIF)

S2 Fig Evaluation of the urinary panel in combination with plasma CA19-9: Control versus late and all stage PDAC samples. Performance in distinguishing control samples (*n* = 92) from late stage PDAC (stage III–IV) (*n* = 70) (A and D) and from PDAC at all stages (*n* = 150) (B and D). ROC curve for CA19-9 and biomarker panel alone and in combination (A and B); summary of the performances (C and D).
(TIF)

S3 Fig Evaluation of the urinary panel in combination with plasma CA19-9: Benign versus late and all stage PDAC samples. Performance in distinguishing benign samples (*n* = 108) from late stage PDAC (stage III–IV) (*n* = 70) (A and D) and from PDAC at all stages (*n* = 150) (B and D). ROC curve of CA19-9 and biomarker panel alone and in combination (A and B); summary of the performances (C and D).
(TIF)

S4 Fig Performance of urine biomarker panel in distinguishing CP from PDAC. Comparison of PDAC stages I–II, III–IV, and I–IV to CP: (A) ROC curve of the 3-biomarker panel in the validation sets (50% of the samples). (B) Performance of the biomarkers in the training and validation sets expressed as area under the ROC curve (AUC). (C) SN of the urinary panel with different SP cutoffs.
(TIF)

S5 Fig Evaluation of the urinary panel in combination with plasma CA19-9: CP versus PDAC samples. Performance in distinguishing CP samples (*n* = 64) from PDAC stage I–II (*n* = 80) (A and D), PDAC stage III–IV (*n* = 70) (B and E), and PDAC at all stages (*n* = 150) (C and F). ROC curve of CA19-9 and biomarker panel alone and in combination (A–C); summary of the performances (D–F).
(TIF)

S6 Fig Comparison between plasma and urine CA19-9. Violin plots showing (A and B) plasma and urine CA19-9 measured by Roche Cobas and (C) urine CA19-9 measured by ELISA (78 samples comprising 22 control, 9 benign, and 47 PDAC I–IV). The number of samples for each group is shown in parentheses. All data were creatinine normalised. Upper bars: Kruskal–Wallis test, Dunn’s multiple comparisons; ns, not significant.
(TIF)

S7 Fig Comparison of LYVE1, REG1B, and TFF1 in matched plasma and urine samples. The performance of the 3 biomarkers in plasma (A) compared to urine (B). The number of samples per group is shown in parentheses. Urine data were creatinine normalised. Upper bars: Kruskal–Wallis test, Dunn’s multiple comparisons; ns, not significant.
(TIF)

S8 Fig Urine biomarker stability test. (A) Daily level variations of LYVE1, REG1B, and TFF1 in urine collected twice a day, in the morning (AM) and in the afternoon (PM) for 2 days, in the afternoon on the third day, and in the morning on the fourth day, in 4 control individuals. (B and C) Level of LYVE1, REG1B, and TFF1 in urine specimens left at room temperature for up to 5 days when collected (B) in sterile tubes without boric acid or (C) in tubes containing boric acid. All data were creatinine normalised. Each colour represents an individual.
(TIF)

S1 Table Sample details and ELISA results for 590 samples. (XLSX)

S2 Table Validation and training datasets. (DOCX)

S3 Table Positive and negative predictive values. (DOCX)

S4 Table CA19-9 measurements in urine and plasma. (DOCX)

S5 Table ELISA results for stability test. (DOCX)

S6 Table ELISA results of 3 urinary tract cancers. (DOCX)

S1 Appendix Flow diagram and analysis plan. (DOCX)

S2 Appendix REMARK checklist. (DOCX)

## Abbreviations

AUC: area under the receiver operating characteristic curve
CI: confidence interval
CP: chronic pancreatitis
FPC: familial pancreatic cancer
NOD: new onset diabetes
PanIN: pancreatic intraepithelial neoplasia
PC: prostate cancer
PDAC: pancreatic ductal adenocarcinoma
RCC: renal cell carcinoma
ROC: receiver operating characteristic
SN: sensitivity
SP: specificity
TCC: transitional cell cancer
