# Supplementary figures and images for: A combination of urinary biomarker panel and PancRISK score for earlier detection of pancreatic cancer: A case–control study
Source: PLoS Med. 2020 Dec 10;17(12):e1003489. doi: 10.1371/journal.pmed.1003489 (PMC7758047; doi:10.1371/journal.pmed.1003489)

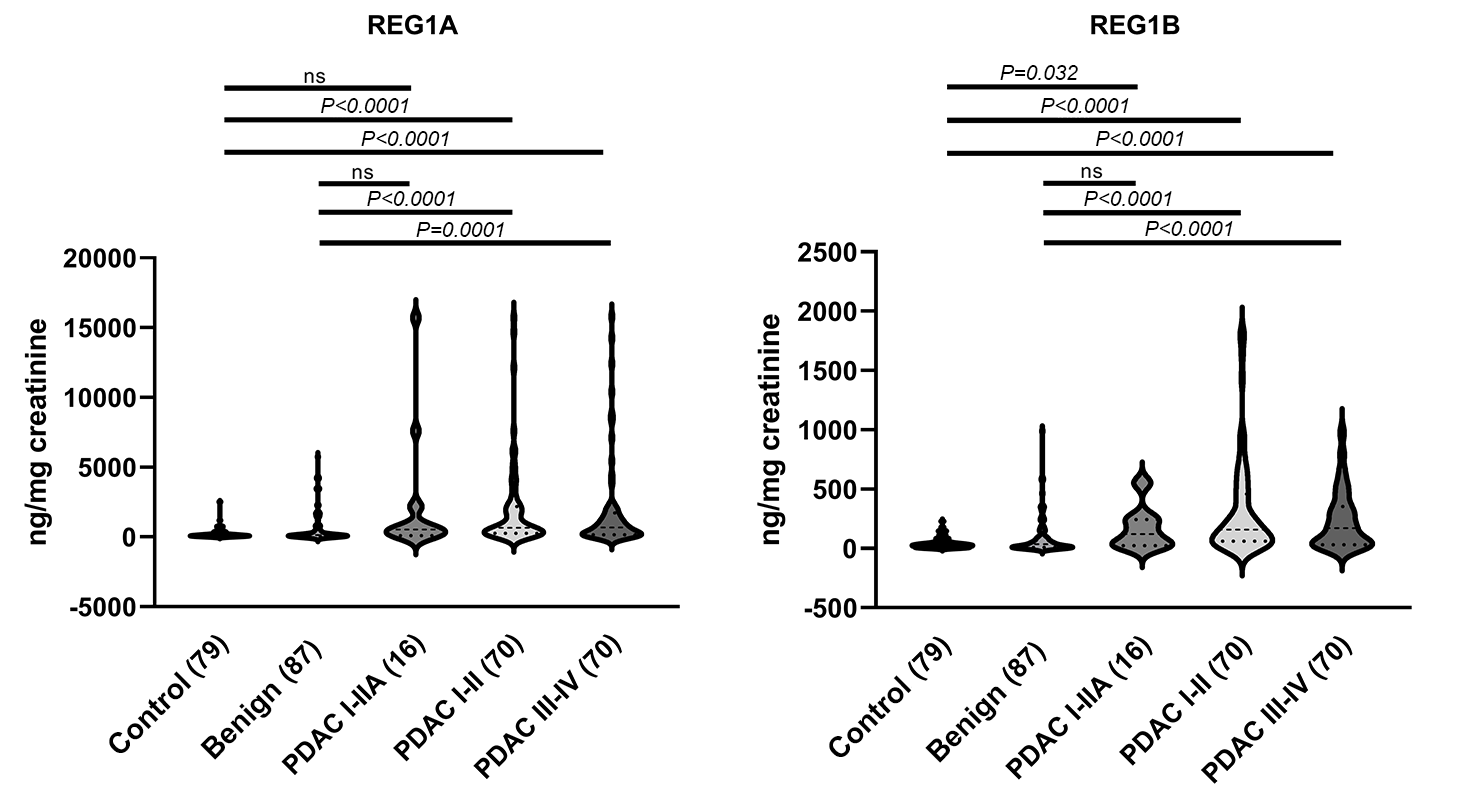

Supplement: S1 Fig — Violin plots showing the levels of REG1A and REG1B in 306 urine samples (79 control, 87 benign, and 140 PDAC [16 stage I–IIA, 70 stage I–II, and 70 stage III–IV]). All data were creatinine normalised. Upper bars: Kruskal–Wallis test, Dunn’s multiple comparisons; ns, not significant. (TIF) [file pmed.1003489.s001.tif]

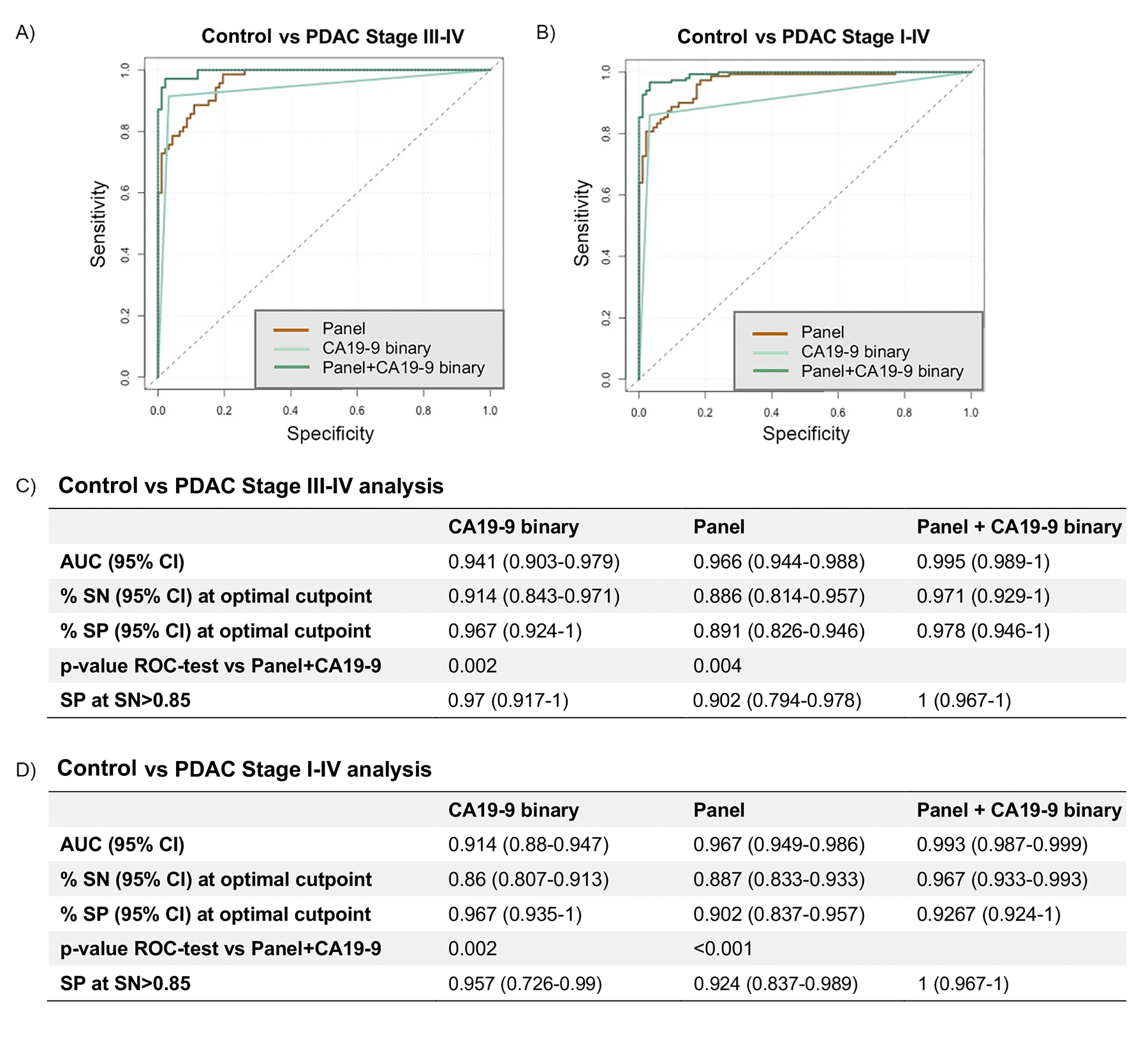

Supplement: S2 Fig — Performance in distinguishing control samples (n = 92) from late stage PDAC (stage III–IV) (n = 70) (A and D) and from PDAC at all stages (n = 150) (B and D). ROC curve for CA19-9 and biomarker panel alone and in combination (A and B); summary of the performances (C and D). (TIF) [file pmed.1003489.s002.tif]

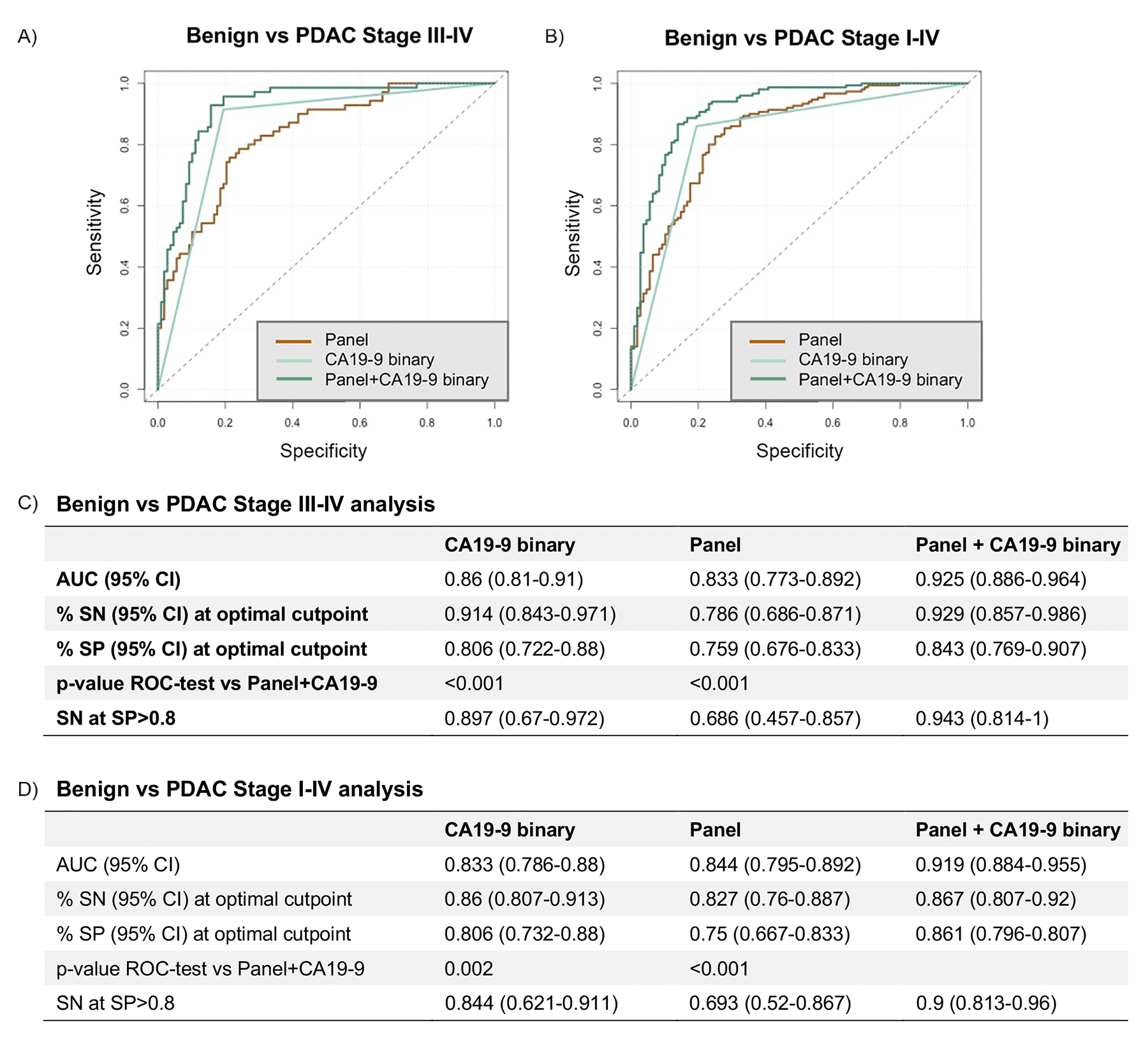

Supplement: S3 Fig — Performance in distinguishing benign samples (n = 108) from late stage PDAC (stage III–IV) (n = 70) (A and D) and from PDAC at all stages (n = 150) (B and D). ROC curve of CA19-9 and biomarker panel alone and in combination (A and B); summary of the performances (C and D). (TIF) [file pmed.1003489.s003.tif]

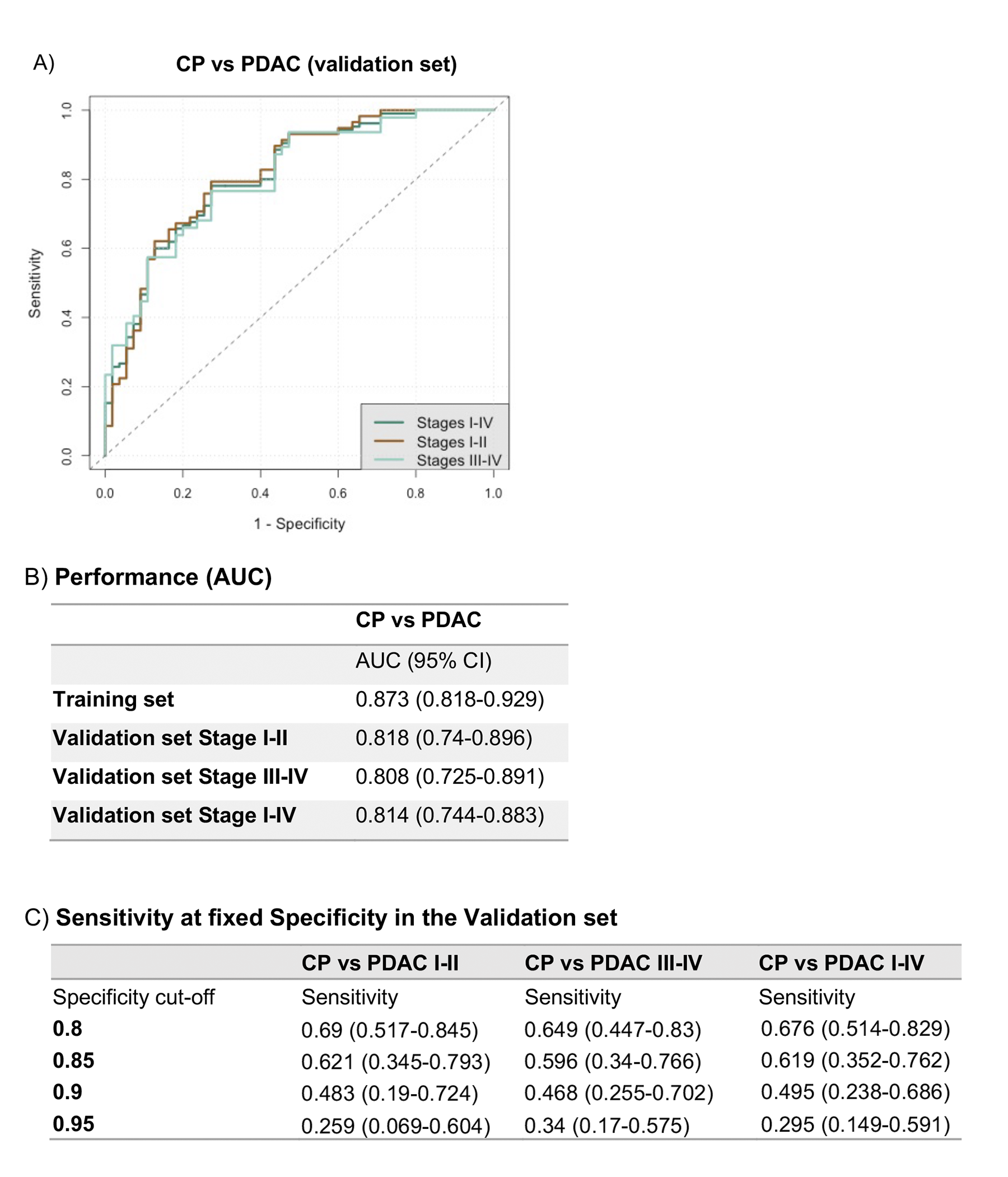

Supplement: S4 Fig — Comparison of PDAC stages I–II, III–IV, and I–IV to CP: (A) ROC curve of the 3-biomarker panel in the validation sets (50% of the samples). (B) Performance of the biomarkers in the training and validation sets expressed as area under the ROC curve (AUC). (C) SN of the urinary panel with different SP cutoffs. (TIF) [file pmed.1003489.s004.tif]

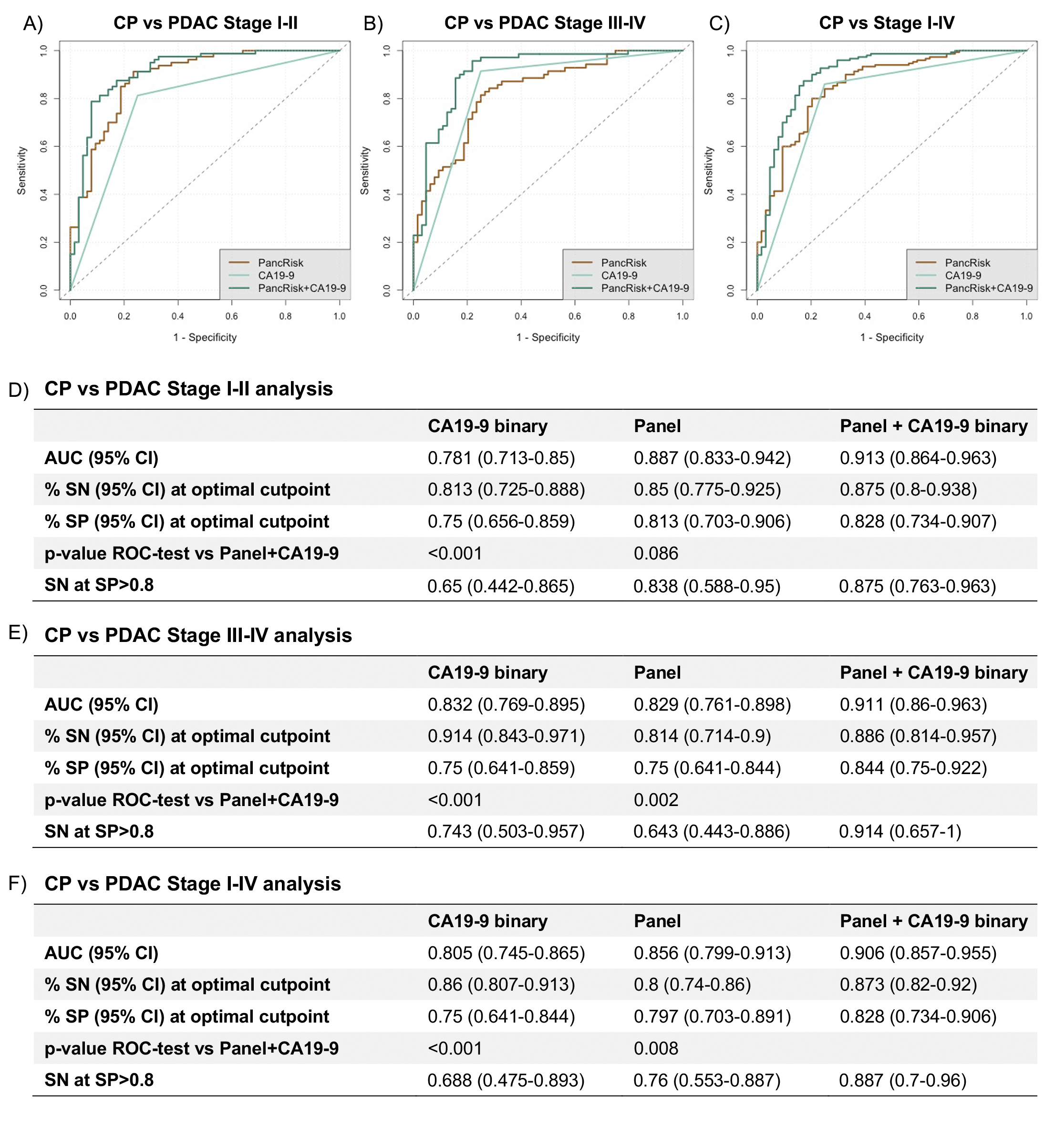

Supplement: S5 Fig — Performance in distinguishing CP samples (n = 64) from PDAC stage I–II (n = 80) (A and D), PDAC stage III–IV (n = 70) (B and E), and PDAC at all stages (n = 150) (C and F). ROC curve of CA19-9 and biomarker panel alone and in combination (A–C); summary of the performances (D–F). (TIF) [file pmed.1003489.s005.tif]

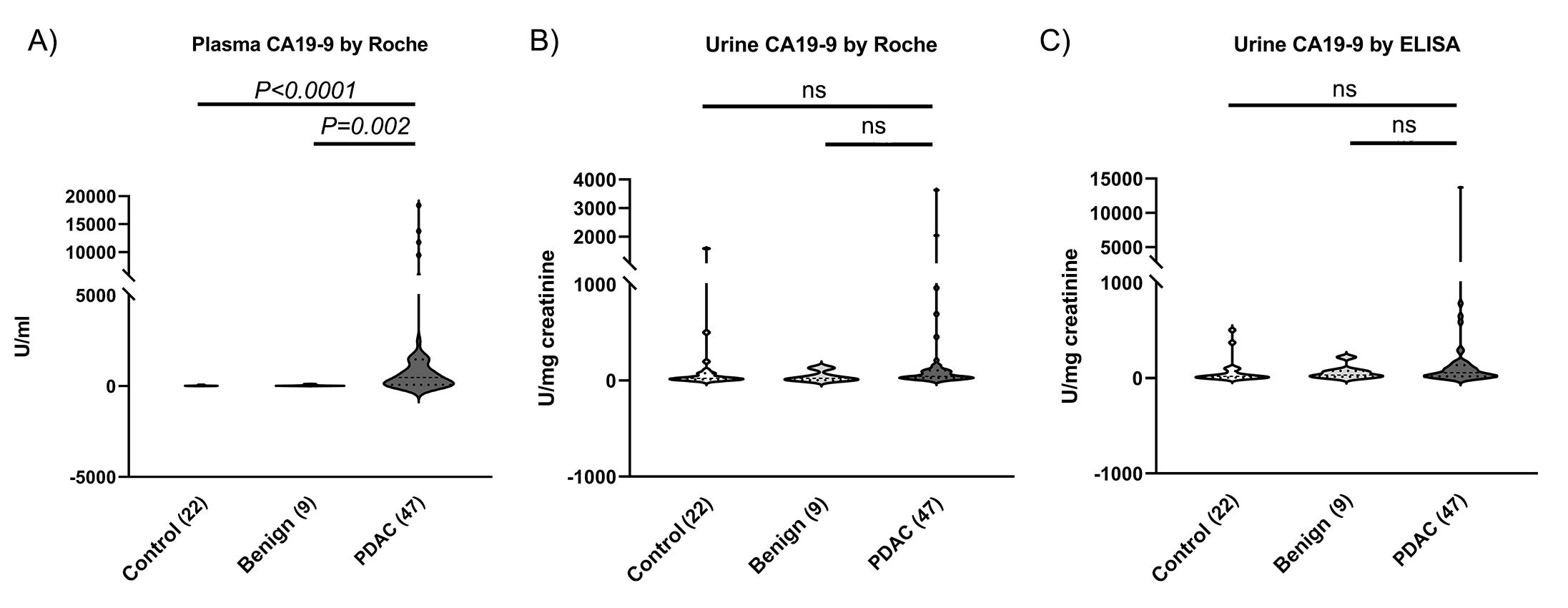

Supplement: S6 Fig — Violin plots showing (A and B) plasma and urine CA19-9 measured by Roche Cobas and (C) urine CA19-9 measured by ELISA (78 samples comprising 22 control, 9 benign, and 47 PDAC I–IV). The number of samples for each group is shown in parentheses. All data were creatinine normalised. Upper bars: Kruskal–Wallis test, Dunn’s multiple comparisons; ns, not significant. (TIF) [file pmed.1003489.s006.tif]

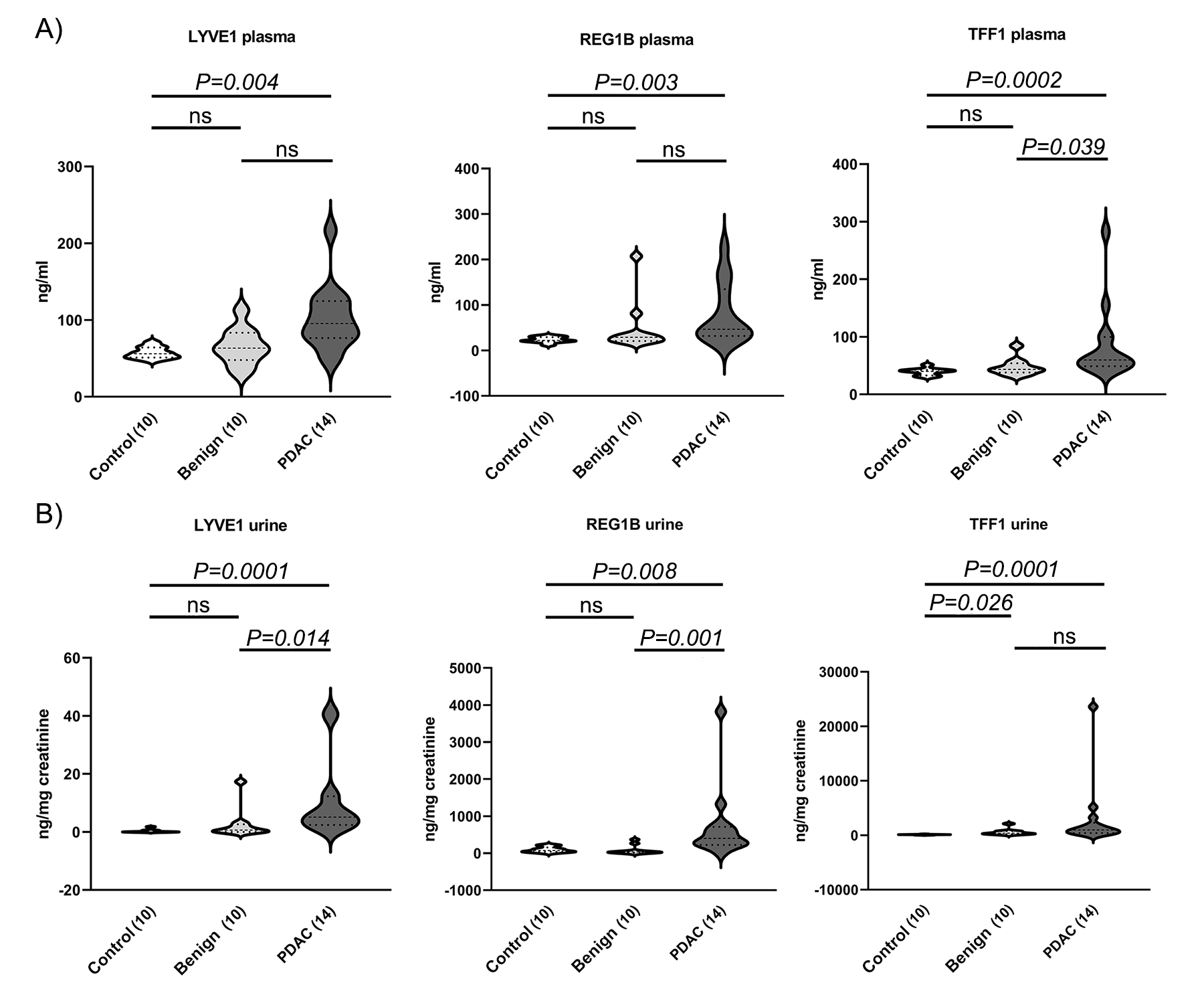

Supplement: S7 Fig — The performance of the 3 biomarkers in plasma (A) compared to urine (B). The number of samples per group is shown in parentheses. Urine data were creatinine normalised. Upper bars: Kruskal–Wallis test, Dunn’s multiple comparisons; ns, not significant. (TIF) [file pmed.1003489.s007.tif]

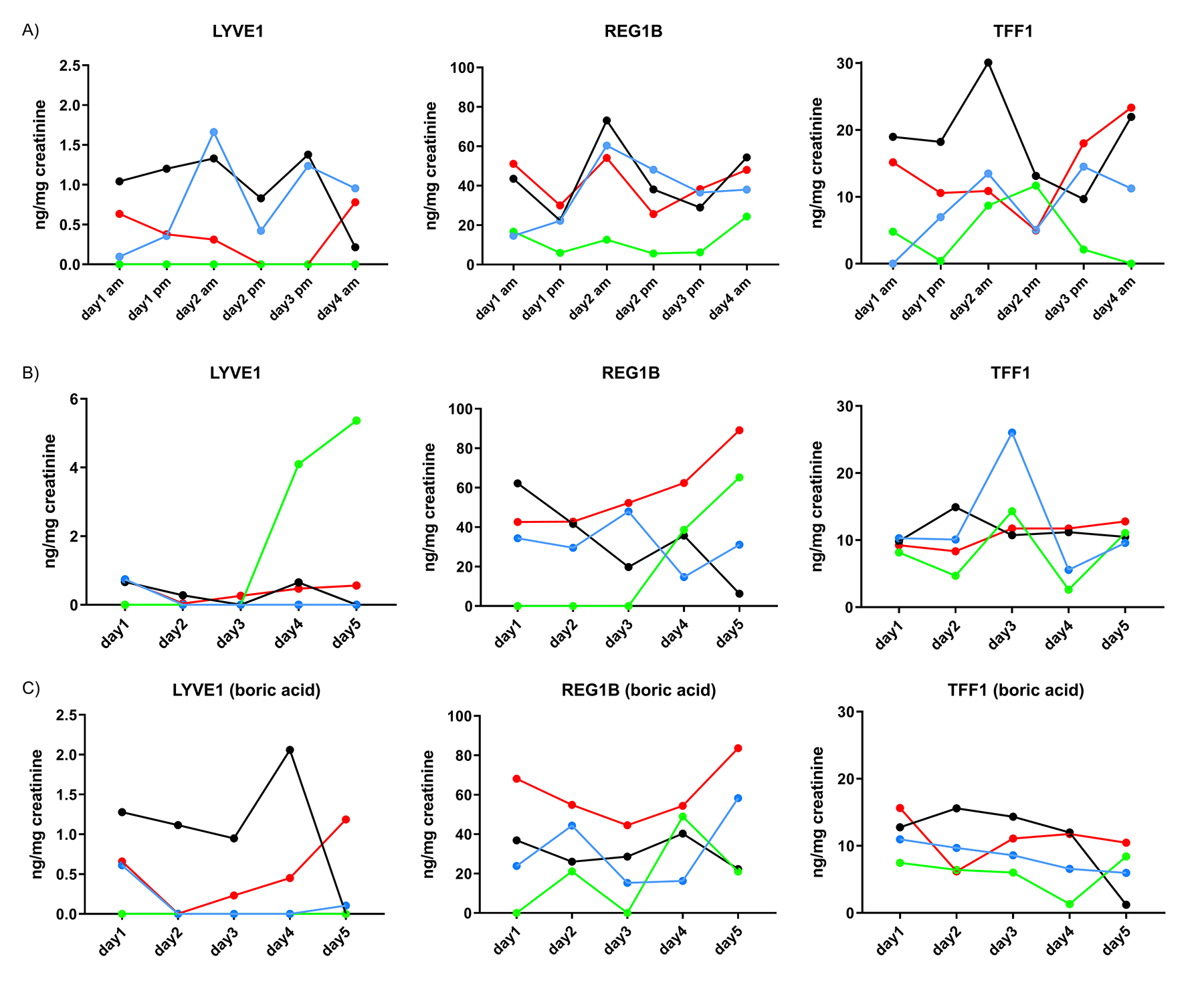

Supplement: S8 Fig — (A) Daily level variations of LYVE1, REG1B, and TFF1 in urine collected twice a day, in the morning (AM) and in the afternoon (PM) for 2 days, in the afternoon on the third day, and in the morning on the fourth day, in 4 control individuals. (B and C) Level of LYVE1, REG1B, and TFF1 in urine specimens left at room temperature for up to 5 days when collected (B) in sterile tubes without boric acid or (C) in tubes containing boric acid. All data were creatinine normalised. Each colour represents an individual. (TIF) [file pmed.1003489.s008.tif]
