# Supplementary material for: A combination of urinary biomarker panel and PancRISK score for earlier detection of pancreatic cancer: A case–control study
Source: PLoS Med. 2020 Dec 10;17(12):e1003489. doi: 10.1371/journal.pmed.1003489 (PMC7758047; doi:10.1371/journal.pmed.1003489)
Supplement: S2 Table — (DOCX) [file pmed.1003489.s010.docx]

**S2 Table. Validation and training datasets.**

(A) Control vs PDAC

|  | Training set (50%): n=187 | Validation set (50%): n=195 |
| --- | --- | --- |
| Control | 84 | 99 |
| PDAC | 103 | 96 |
| Stage I (I, IA, IB) | 7 (1, 2, 4) | 9 (0, 1, 8) |
| Stage II (II, IIA, IIB) | 45 (6, 4, 35) | 41 (1, 7, 33) |
| Stage III | 41 | 35 |
| Stage IV | 10 | 11 |

(B) Benign vs PDAC

|  | Training set (50%): n=209 | Validation set (50%): n=198 |
| --- | --- | --- |
| Benign | 112 | 96 |
| PDAC | 97 | 102 |
| Stage I (I, IA, IB) | 6 (0, 1, 5) | 10 (1, 2, 7) |
| Stage II (II, IIA, IIB) | 42 (2, 5, 35) | 44 (5, 6, 33) |
| Stage III | 35 | 41 |
| Stage IV | 14 | 7 |

Details and number of samples in each group.
