# Supplementary material for: A combination of urinary biomarker panel and PancRISK score for earlier detection of pancreatic cancer: A case–control study
Source: PLoS Med. 2020 Dec 10;17(12):e1003489. doi: 10.1371/journal.pmed.1003489 (PMC7758047; doi:10.1371/journal.pmed.1003489)
Supplement: S3 Table — (DOCX) [file pmed.1003489.s011.docx]

**S3 Table**. **Positive and negative predictive values.**

A) Control vs PDAC

| **Prevalence** | **Control versus PDAC I-II** | | | | **Control versus PDAC I-IV** | | | |
| --- | --- | --- | --- | --- | --- | --- | --- | --- |
|  | **PancRisk** | | | | **PancRisk** | | | |
|  | **SN** | **SP** | **PPV** | **NPV** | **SN** | **SP** | **PPV** | **NPV** |
| **0.01** | 0.85 | 0.919 (0.677-0.98) | 0.096 | 0.998 | 0.85 | 0.828 (0.657-0.96) | 0.048 | 0.998 |
| **0.015** | 0.85 | 0.919 (0.677-0.98) | 0.138 | 0.998 | 0.85 | 0.828 (0.657-0.96) | 0.070 | 0.997 |
| **0.02** | 0.85 | 0.919 (0.677-0.98) | 0.176 | 0.997 | 0.85 | 0.828 (0.657-0.96) | 0.092 | 0.996 |
| **0.025** | 0.85 | 0.919 (0.677-0.98) | 0.212 | 0.996 | 0.85 | 0.828 (0.657-0.96) | 0.112 | 0.995 |
| **0.03** | 0.85 | 0.919 (0.677-0.98) | 0.245 | 0.995 | 0.85 | 0.828 (0.657-0.96) | 0.133 | 0.994 |

PPV, positive predictive value; NPV, negative predictive value; SN, sensitivity; SP, specificity.

B) Benign vs PDAC

| **Prevalence** | **Benign versus PDAC I-II** | | | | **Benign versus PDAC I-IV** | | | |
| --- | --- | --- | --- | --- | --- | --- | --- | --- |
|  | **PancRisk** | | | | **PancRisk** | | | |
|  | **SN** | **SP** | **PPV** | **NPV** | **SN** | **SP** | **PPV** | **NPV** |
| **0.01** | 0.796 (0.574-0.944) | 0.8 | 0.039 | 0.997 | 0.725 (0.569-0.853) | 0.8 | 0.035 | 0.997 |
| **0.015** | 0.796 (0.574-0.944) | 0.8 | 0.057 | 0.996 | 0.725 (0.569-0.853) | 0.8 | 0.052 | 0.995 |
| **0.02** | 0.796 (0.574-0.944) | 0.8 | 0.075 | 0.995 | 0.725 (0.569-0.853) | 0.8 | 0.069 | 0.993 |
| **0.025** | 0.796 (0.574-0.944) | 0.8 | 0.093 | 0.994 | 0.725 (0.569-0.853) | 0.8 | 0.085 | 0.991 |
| **0.03** | 0.796 (0.574-0.944) | 0.8 | 0.110 | 0.992 | 0.725 (0.569-0.853) | 0.8 | 0.101 | 0.989 |

PPV, positive predictive value; NPV, negative predictive value; SN, sensitivity; SP, specificity.
