# Supplementary material for: A combination of urinary biomarker panel and PancRISK score for earlier detection of pancreatic cancer: A case–control study
Source: PLoS Med. 2020 Dec 10;17(12):e1003489. doi: 10.1371/journal.pmed.1003489 (PMC7758047; doi:10.1371/journal.pmed.1003489)
Supplement: S4 Table — (DOCX) [file pmed.1003489.s012.docx]

**S4 Table. Plasma and urine CA19-9 data measured by Roche Cobas and ELISA.**

| **Sample ID** | **Diagnosis (1=Control, 2= Benign, 3=PDAC)** | **Stage** | **Plasma CA19-9 U/ml (Roche)** | **Urine CA19-9 U/ml (Roche)** | **Urine CA19-9 U/ml (ELISA)** |
| --- | --- | --- | --- | --- | --- |
| **S1** | 1 |  | 11.7 | 19.75 | 37.244 |
| **S26** | 1 |  | 6.94 | 15 | 19.36 |
| **S27** | 1 |  | 8.47 | 5 | 0 |
| **S30** | 1 |  | 13.58 | 5 | 5.634 |
| **S31** | 1 |  | 7.86 | 36 | 22.04 |
| **S33** | 1 |  | 3.53 | 1 | 0 |
| **S34** | 1 |  | 6.58 | 9 | 8.941 |
| **S37** | 1 |  | 5.8 | 38 | 74.487 |
| **S41** | 1 |  | 3.59 | 6 | 0 |
| **S42** | 1 |  | 7.55 | 7 | 6.641 |
| **S45** | 1 |  | 5.7 | 28 | 19.628 |
| **S46** | 1 |  | 37.03 | 98 | 47.23 |
| **S48** | 1 |  | 12.84 | 6 | 0 |
| **S53** | 1 |  | 6.77 | 17 | 3.98 |
| **S54** | 1 |  | 10.43 | 43 | 22.308 |
| **S55** | 1 |  | 7.57 | 16 | 8.941 |
| **S56** | 1 |  | 31.24 | 69 | 36.029 |
| **S57** | 1 |  | 28.18 | 33 | 19.092 |
| **S58** | 1 |  | 18.23 | 436 | 324.114 |
| **S75** | 1 |  | 84.3 | 22.11 | 37.244 |
| **S76** | 1 |  | 0.6 | 0.5 | 19.123 |
| **C77** | 1 |  | 29.37 | 1645 | 524.577 |
| **S233** | 2 |  | 114.8 | 71.16 | 124.8 |
| **S239** | 2 |  | 32.3 | 1.24 | 12.312 |
| **S244** | 2 |  | 15.2 | 1.46 | 0 |
| **S249** | 2 |  | 19 | 13.42 | 36.85 |
| **S252** | 2 |  | 46.1 | 96.78 | 168.791 |
| **S254** | 2 |  | 16.2 | 55.13 | 60.253 |
| **S286** | 2 |  | 10.9 | 197.4 | 88.6 |
| **S294** | 2 |  | 7.9 | 9.8 | 0 |
| **S307** | 2 |  | 6.9 | 4.51 | 22.289 |
| **S531** | 3 | IB | 55.6 | 9.84 | 11.077 |
| **S404** | 3 | IIB | 554 | 7 | 0 |
| **S407** | 3 | IIB | 33 | 21 | 12.662 |
| **S421** | 3 | IIB | 941 | 21 | 16.681 |
| **S422** | 3 | IIB | 74 | 28 | 13.075 |
| **S423** | 3 | IIB | 91 | 45 | 16.145 |
| **S425** | 3 | IIB | 35 | 64 | 32.033 |
| **S429** | 3 | IIB | 600 | 43 | 17.752 |
| **S431** | 3 | IIB | 1510 | 72 | 20.164 |
| **S432** | 3 | IIB | 1465 | 14 | 0 |
| **S433** | 3 | IIB | 1503 | 87 | 26.014 |
| **S440** | 3 | IIB | 110 | 37 | 24.967 |
| **S443** | 3 | IIB | 64 | 5 | 0 |
| **S447** | 3 | IIB | 100 | 19 | 15.609 |
| **S485** | 3 | IIB | 248.8 | 10 | 39.216 |
| **S525** | 3 | IIB | 523 | 9.72 | 27 |
| **S537** | 3 | IIB | 158 | 42.33 | 38.6 |
| **S541** | 3 | IIB | 1749 | 1397 | 5280 |
| **S547** | 3 | IIB | 15 | 0.6 | 11.077 |
| **S557** | 3 | IIB | 41 | 1.43 | 23.872 |
| **S567** | 3 | IIB | 11740 | 121 | 203.733 |
| **S479** | 3 | III | 9436 | 51.89 | 89.7 |
| **P480** | 3 | III | 330.8 | 59.59 | 68.1 |
| **S481** | 3 | III | 641 | 187 | 150.27 |
| **P483** | 3 | III | 5731 | 461.1 | 597.1 |
| **S486** | 3 | III | 69.75 | 90 | 83.492 |
| **S487** | 3 | III | 783.2 | 38 | 62.835 |
| **S491** | 3 | III | 1318 | 74 | 169.112 |
| **S492** | 3 | III | 3.04 | 1 | 17.54 |
| **S494** | 3 | III | 16.92 | 22 | 45.13 |
| **S523** | 3 | III | 147.7 | 31.53 | 12.5 |
| **S528** | 3 | III | 2484 | 250.7 | 152.3 |
| **S532** | 3 | III | 1555 | 21.39 | 25.905 |
| **S533** | 3 | III | 422 | 128.1 | 183.4 |
| **S542** | 3 | III | 57 | 10.48 | 19.5 |
| **S550** | 3 | III | 885 | 34.73 | 50.86 |
| **S562** | 3 | III | 468.3 | 61 | 54.673 |
| **S563** | 3 | III | 18360 | 48 | 61.729 |
| **S565** | 3 | III | 433 | 8 | 0 |
| **S568** | 3 | III | 14.67 | 9 | 51.623 |
| **S571** | 3 | III | 633 | 1384 | 1566.332 |
| **S574** | 3 | III | 84 | 94 | 115.603 |
| **S576** | 3 | III | 99.6 | 21 | 46.666 |
| **S495** | 3 | IV | 710.8 | 7 | 26.313 |
| **S519** | 3 | IV | 941 | 10.56 | 6.3 |
| **S529** | 3 | IV | 13740 | 39.67 | 2.7 |
| **S590** | 3 | IV | 1488 | 40.5 | 0 |
