# Supplementary material for: A combination of urinary biomarker panel and PancRISK score for earlier detection of pancreatic cancer: A case–control study
Source: PLoS Med. 2020 Dec 10;17(12):e1003489. doi: 10.1371/journal.pmed.1003489 (PMC7758047; doi:10.1371/journal.pmed.1003489)
Supplement: S5 Table — (DOCX) [file pmed.1003489.s013.docx]

**S5 Table. Daily variation in the concentration and the stability of the three proteins in urine in four control subject donors.**

A) Urinary concentration of the three biomarkers collected twice a day.

| **Sample ID** | **Timepoint** | **LYVE1 ng/ml** | **REG1B ng/ml** | **TFF1 ng/ml** | **Creatinine mg/ml** |
| --- | --- | --- | --- | --- | --- |
| **Sa** | day1_am | 0.0241961 | 3.6479925 | 0 | 0.24882 |
| **Sa** | day1_pm | 0.3845164 | 23.891955 | 7.470892 | 1.07445 |
| **Sb** | day1_am | 0 | 3.4033525 | 0.9751627 | 0.20358 |
| **Sb** | day1_pm | 0 | 1.0792725 | 0.0750125 | 0.18096 |
| **Sc** | day1_am | 2.276198 | 94.95985 | 41.40911 | 2.18283 |
| **Sc** | day1_pm | 2.417753 | 45.11447 | 36.67935 | 2.01318 |
| **Sd** | day1_am | 0.6032824 | 48.60059 | 14.40066 | 0.95004 |
| **Sd** | day1_pm | 0.3973851 | 31.59811 | 11.11318 | 1.05183 |
| **Sa** | day2_am | 1.25958 | 45.72607 | 10.2158 | 0.75777 |
| **Sa** | day2_pm | 0.1142762 | 13.06663 | 1.375229 | 0.27144 |
| **Sb** | day2_am | 0 | 1.9966725 | 1.375229 | 0.15834 |
| **Sb** | day2_pm | 0 | 1.1404325 | 2.375396 | 0.20358 |
| **Sc** | day2_am | 1.761455 | 96.79465 | 39.80956 | 1.32327 |
| **Sc** | day2_pm | 0.8735227 | 40.09935 | 13.81147 | 1.05183 |
| **Sd** | day2_am | 0.2815678 | 48.96755 | 9.846294 | 0.9048 |
| **Sd** | day2_pm | 0 | 12.760835 | 2.475413 | 0.49764 |
| **Sa** | day3_pm | 1.594164 | 47.13275 | 18.70176 | 1.28934 |
| **Sb** | day3_pm | 0 | 1.6908725 | 0.5750959 | 0.27144 |
| **Sc** | day3_pm | 1.808976 | 38.019915 | 12.69412 | 1.31196 |
| **Sd** | day3_pm | 0 | 48.96755 | 22.98971 | 1.27803 |
| **Sa** | day4_am | 1.015077 | 40.40515 | 11.95776 | 1.06314 |
| **Sb** | day4_am | 0 | 3.5868325 | 0 | 0.14703 |
| **Sc** | day4_am | 0.2686992 | 67.6825 | 27.3302 | 1.2441 |
| **Sd** | day4_am | 1.182369 | 72.86505 | 35.3593 | 1.51554 |

B) Urinary concentration of the three biomarkers kept at room temperature for up to five days in absence/presence of boric acid.

| **Sample ID** | **Timepoint** | **Collection tube** | **LYVE1 ng/ml** | **REG1B ng/ml** | **TFF1 ng/ml** | **Creatinine mg/ml** |
| --- | --- | --- | --- | --- | --- | --- |
| **Sa** | day1 | untreated tube | 1.015775 | 47.010435 | 14.06879 | 1.36851 |
| **Sb** | day1 | untreated tube | 0 | 0 | 4.153525 | 0.50895 |
| **Sc** | day1 | untreated tube | 0.4984708 | 46.45999 | 7.336811 | 0.74646 |
| **Sd** | day1 | untreated tube | 0.693897 | 40.95559 | 8.885249 | 0.96135 |
| **Sa** | day2 | untreated tube | 0 | 20.03887 | 6.850297 | 0.6786 |
| **Sb** | day2 | untreated tube | 0 | 0 | 1.325454 | 0.28275 |
| **Sc** | day2 | untreated tube | 0.4294969 | 64.0129 | 22.92216 | 1.53816 |
| **Sd** | day2 | untreated tube | 0.03864456 | 44.07475 | 8.575516 | 1.02921 |
| **Sa** | day3 | untreated tube | 0 | 26.582995 | 14.42379 | 0.55419 |
| **Sb** | day3 | untreated tube | 0 | 0 | 2.916125 | 0.20358 |
| **Sc** | day3 | untreated tube | 0 | 3.5868325 | 1.944019 | 0.18096 |
| **Sd** | day3 | untreated tube | 0.291549 | 58.56965 | 13.13744 | 1.11969 |
| **Sa** | day4 | untreated tube | 0 | 11.78227 | 4.462923 | 0.80301 |
| **Sb** | day4 | untreated tube | 0.5559491 | 5.238155 | 0.3534509 | 0.13572 |
| **Sc** | day4 | untreated tube | 1.257184 | 69.0972 | 21.62879 | 1.93401 |
| **Sd** | day4 | untreated tube | 0.4984708 | 65.6382 | 12.33953 | 1.05183 |
| **Sa** | day5 | untreated tube | 0 | 12.33271 | 3.799962 | 0.39585 |
| **Sb** | day5 | untreated tube | 0.7283839 | 8.84659 | 1.502187 | 0.13572 |
| **Sc** | day5 | untreated tube | 0 | 1.1404325 | 1.899836 | 0.18096 |
| **Sd** | day5 | untreated tube | 0.6364187 | 100.846 | 14.46816 | 1.131 |
| **Sa** | day1 | boric acid | 0.8088535 | 31.59811 | 14.51254 | 1.32327 |
| **Sb** | day1 | boric acid | 0 | 0 | 3.623192 | 0.48633 |
| **Sc** | day1 | boric acid | 1.199706 | 34.59495 | 11.98494 | 0.93873 |
| **Sd** | day1 | boric acid | 0.4180013 | 43.15735 | 9.903272 | 0.63336 |
| **Sa** | day2 | boric acid | 0 | 28.11199 | 6.14278 | 0.63336 |
| **Sb** | day2 | boric acid | 0 | 5.972075 | 1.81147 | 0.28275 |
| **Sc** | day2 | boric acid | 1.71701 | 40.09935 | 23.99339 | 1.53816 |
| **Sd** | day2 | boric acid | 0.004157587 | 55.26705 | 6.23122 | 1.00659 |
| **Sa** | day3 | boric acid | 0 | 21.26207 | 11.94064 | 1.39113 |
| **Sb** | day3 | boric acid | 0 | 0 | 2.518443 | 0.41847 |
| **Sc** | day3 | boric acid | 2.027393 | 61.19955 | 30.66171 | 2.13759 |
| **Sd** | day3 | boric acid | 0.257062 | 49.39567 | 12.2952 | 1.10838 |
| **Sa** | day4 | boric acid | 0 | 13.80055 | 5.568031 | 0.84825 |
| **Sb** | day4 | boric acid | 0 | 6.64483 | 0.1767255 | 0.13572 |
| **Sc** | day4 | boric acid | 3.88969 | 76.13875 | 22.65451 | 1.88877 |
| **Sd** | day4 | boric acid | 0.4754795 | 57.176 | 12.38385 | 1.05183 |
| **Sa** | day5 | boric acid | 0.05014021 | 27.68387 | 2.827751 | 0.47502 |
| **Sb** | day5 | boric acid | 0 | 7.868035 | 3.137076 | 0.37323 |
| **Sc** | day5 | boric acid | 0 | 3.2810325 | 0.1767255 | 0.14703 |
| **Sd** | day5 | boric acid | 1.153723 | 81.3273 | 10.16892 | 0.97266 |
