# Supplementary material for: A combination of urinary biomarker panel and PancRISK score for earlier detection of pancreatic cancer: A case–control study
Source: PLoS Med. 2020 Dec 10;17(12):e1003489. doi: 10.1371/journal.pmed.1003489 (PMC7758047; doi:10.1371/journal.pmed.1003489)
Supplement: S6 Table — (DOCX) [file pmed.1003489.s014.docx]

**S6 Table. Details and ELISA results of three urinary tract cancers.**

| **Sample ID** | **Age** | **Sex** | **Diagnosis** | **Type** | **Creatinine mg/l** | **LYVE1 ng/ml** | **REG1B ng/ml** | **TFF1 ng/ml** |
| --- | --- | --- | --- | --- | --- | --- | --- | --- |
| **3136** | 69 | M | Prostate Cancer |  | 0.396 | 0.074 | 107.436 | 236.728 |
| **3137** | 83 | M | Prostate Cancer |  | 0.283 | 0.004 | 3.731 | 52.099 |
| **3138** | 74 | M | Prostate Cancer |  | 0.407 | 0.014 | 40.145 | 81.522 |
| **3139** | 69 | M | Prostate Cancer |  | 0.713 | 0.531 | 97.319 | 185.238 |
| **3140** | 73 | M | Prostate Cancer |  | 0.260 | 0.130 | 4.060 | 24.883 |
| **3141** | 52 | M | Prostate Cancer |  | 0.305 | 0.022 | 12.102 | 6.494 |
| **3142** | 53 | M | Prostate Cancer |  | 0.396 | 0.037 | 57.035 | 63.133 |
| **3144** | 58 | M | Prostate Cancer |  | 1.606 | 0.027 | 5.215 | 77.844 |
| **3146** | 72 | M | Prostate Cancer |  | 1.063 | 0.563 | 169.520 | 814.153 |
| **3147** | 58 | M | Prostate Cancer |  | 0.215 | 0.009 | 16.214 | 21.205 |
| **3148** | 66 | M | Prostate Cancer |  | 0.961 | 0.447 | 97.086 | 340.444 |
| **3150** | 68 | M | Prostate Cancer |  | 1.142 | 0.406 | 99.879 | 549.347 |
| **3152** | 71 | M | Prostate Cancer |  | 0.769 | 0.000 | 4.459 | 77.844 |
| **3158** | 56 | M | Prostate Cancer |  | 0.079 | 0.004 | 0.000 | 8.700 |
| **3159** | 77 | M | Prostate Cancer |  | 0.430 | 0.052 | 8.061 | 44.008 |
| **3160** | 73 | M | Prostate Cancer |  | 0.283 | 0.012 | 3.822 | 32.239 |
| **3162** | 66 | M | Prostate Cancer |  | 0.452 | 0.065 | 17.118 | 80.051 |
| **3163** | 75 | M | Prostate Cancer |  | 0.215 | 0.140 | 45.852 | 122.714 |
|  |  |  |  |  |  |  |  |  |
| **6282** | 81 | M | Renal Cell Carcinoma |  | 1.176 | 9.947 | 131.607 | 3088.356 |
| **6285** | 67 | M | Renal Cell Carcinoma |  | 0.385 | 6.328 | 12.665 | 578.096 |
| **6289** | 67 | M | Renal Cell Carcinoma |  | 1.074 | 12.084 | 73.587 | 1801.431 |
| **6290** | 80 | M | Renal Cell Carcinoma |  | 0.916 | 11.485 | 12.537 | 1142.324 |
| **6292** | 60 | M | Renal Cell Carcinoma |  | 0.746 | 0.354 | 2.597 | 37.083 |
| **6294** | 60 | M | Renal Cell Carcinoma |  | 2.511 | 9.485 | 12.537 | 2000.274 |
| **6295** | 77 | M | Renal Cell Carcinoma |  | 0.554 | 6.505 | 53.579 | 1420.905 |
| **6297** | 71 | M | Renal Cell Carcinoma |  | 0.984 | 14.288 | 50.693 | 3255.908 |
| **6298** | 70 | M | Renal Cell Carcinoma |  | 1.074 | 11.771 | 103.626 | 2668.465 |
| **6299** | 55 | M | Renal Cell Carcinoma |  | 0.916 | 2.844 | 7.984 | 798.135 |
| **6300** | 63 | F | Renal Cell Carcinoma |  | 0.441 | 2.450 | 6.829 | 552.862 |
| **6301** | 56 | M | Renal Cell Carcinoma |  | 0.113 | 0.966 | 5.739 | 24.971 |
| **6302** | 20 | F | Renal Cell Carcinoma |  | 0.792 | 0.980 | 10.356 | 611.405 |
| **6303** | 74 | F | Renal Cell Carcinoma |  | 0.882 | 3.688 | 3.238 | 1763.076 |
| **6304** | 66 | M | Renal Cell Carcinoma |  | 0.611 | 0.994 | 0.224 | 121.868 |
| **7021** | 78 | M | Renal Cell Carcinoma |  | 2.386 | 6.505 | 17.795 | 1762.066 |
| **7025** | 74 | M | Renal Cell Carcinoma |  | 1.018 | 4.586 | 25.042 | 674.994 |
| **7026** | 72 | M | Renal Cell Carcinoma |  | 0.814 | 10.968 | 13.819 | 1082.772 |
| **7028** | 61 | M | Renal Cell Carcinoma |  | 0.226 | 0.340 | 14.909 | 196.561 |
| **7029** | 48 | M | Renal Cell Carcinoma |  | 1.233 | 12.356 | 48.384 | 2805.737 |
| **7030** | 85 | M | Renal Cell Carcinoma |  | 0.758 | 17.091 | 138.586 | 3317.479 |
| **7031** | 68 | M | Renal Cell Carcinoma |  | 0.916 | 16.751 | 124.750 | 3256.918 |
| **7033** | 70 | F | Renal Cell Carcinoma |  | 1.007 | 10.451 | 67.687 | 2518.071 |
| **7035** | 54 | F | Renal Cell Carcinoma |  | 0.351 | 2.722 | 141.428 | 1403.746 |
| **7036** | 65 | M | Renal Cell Carcinoma |  | 1.425 | 6.083 | 58.196 | 1113.053 |
| **7042** | 45 | M | Renal Cell Carcinoma |  | 1.199 | 9.389 | 6.637 | 972.753 |
| **7043** | 72 | M | Renal Cell Carcinoma |  | 0.973 | 6.627 | 8.753 | 905.126 |
| **7044** | 65 | M | Renal Cell Carcinoma |  | 1.866 | 3.810 | 15.807 | 730.508 |
| **7046** | 65 | F | Renal Cell Carcinoma |  | 1.221 | 7.117 | 3.366 | 580.115 |
|  |  |  |  |  |  |  |  |  |
| **U1** | 56 | F | Bladder Cancer | TCC | 0.317 | 0.686 | 2.677 | 14.826 |
| **U2** | 67 | M | Bladder Cancer | TCC | 0.905 | 3.569 | 63.985 | 53.833 |
| **U3** | 60 | M | Bladder Cancer | TCC | 0.509 | 1.477 | 2.436 | 21.655 |
| **U4** | 80 | F | Bladder Cancer | TCC | 1.188 | 2.198 | 85.025 | 133.177 |
| **U5** | 81 | M | Bladder Cancer | TCC | 0.588 | 3.181 | 15.389 | 4.353 |
| **U6** | 74 | F | Bladder Cancer | TCC | 0.995 | 1.251 | 0.899 | 41.066 |
| **U7** | 44 | M | Bladder Cancer | TCC | 0.724 | 2.425 | 10.509 | 58.087 |
| **U8** | 69 | M | Bladder Cancer | TCC | 1.086 | 3.885 | 88.258 | 24.672 |
| **U9** | 57 | M | Bladder Cancer | TCC | 0.577 | 2.434 | 92.367 | 26.482 |
| **U10** | 64 | F | Bladder Cancer | TCC | 0.826 | 5.572 | 71.555 | 15.182 |
| **U11** | 76 | M | Bladder Cancer | TCC | 0.430 | 2.429 | 10.118 | 11.269 |
| **U12** | 68 | M | Bladder Cancer | TCC | 1.561 | 1.177 | 6.860 | 32.165 |
| **U13** | 66 | F | Bladder Cancer | TCC | 0.939 | 1.693 | 12.868 | 73.389 |
| **U14** | 59 | M | Bladder Cancer | TCC | 0.780 | 0.158 | 1.297 | 8.047 |
| **U15** | 69 | M | Bladder Cancer | TCC | 1.629 | 1.083 | 11.623 | 108.557 |
| **U16** | 58 | F | Bladder Cancer | TCC | 0.441 | 0.057 | 1.152 | 8.574 |
| **U17** | 67 | F | Bladder Cancer | TCC | 0.226 | 2.443 | 63.560 | 6.991 |
| **U18** | 65 | F | Bladder Cancer | TCC | 0.430 | 1.342 | 17.698 | 17.317 |
| **U19** | 64 | F | Bladder Cancer | TCC | 1.516 | 3.008 | 17.158 | 45.907 |
| **U20** | 64 | M | Bladder Cancer | TCC | 0.317 | 0.000 | 3.893 | 14.826 |
| TCC, Transitional Cell Carcinoma | | | |  |  |  |  |  |
