# Supplementary material for: A combination of urinary biomarker panel and PancRISK score for earlier detection of pancreatic cancer: A case–control study
Source: PLoS Med. 2020 Dec 10;17(12):e1003489. doi: 10.1371/journal.pmed.1003489 (PMC7758047; doi:10.1371/journal.pmed.1003489)
Supplement: S1 Appendix — (DOCX) [file pmed.1003489.s015.docx]

| **S1 Appendix. Flow diagram and analysis plan.**   \|  \| **Urine specimens** \| **Plasma specimens** \| \| --- \| --- \| --- \| \| **Sample selection** \| \| \| \| Inclusion  criteria \| 1) Control subjects with no known pancreatic conditions or malignancies, or history of renal diseases at the moment of collection.  2) Patients with benign hepatobiliary diseases (including chronic pancreatitis, gallbladder diseases, cystic lesions of the pancreas and patients with abdominal pain and gastrointestinal symptoms suggestive of pancreatic origin).  3) PDAC patients stages I-IV before surgery or chemotherapeutic treatment.  4) (1), (2), and (3) Age and gender matched wherever possible. \| \| \| 5) Urinary tract cancers (prostate cancer, renal cell carcinoma, bladder transitional cell carcinoma). \|  \| \| Exclusion  criteria \| Current or prior treatment for cancer (chemotherapy, radiotherapy, surgical resection, biological therapy, and immunotherapy) for any malignancy within 5 years of enrolment. \| \| \| Samples  for biomarker training and validation \| **N=590** (183 Control subjects; 208 Benign hepatobiliary diseases; 199 PDAC I-IV (27 I-IIA; 75 IIB; 97 III-IV)). \| **N=350** (92 Control subjects; 108 Benign hepatobiliary diseases; 150 PDAC I-IV (20 I-IIA; 60 IIB; 70 III-IV)). \| \| Samples for stability test \| **N=64** from 4 control subjects. \|  \| \| Urinary tract cancer samples \| **N=67** (18 prostate cancers (PC); 29 renal cell carcinomas (RCC); 20 transitional bladder cancers (TCC)). \|  \| \| **Assay** \| Urine LYVE1, REG1B, TFF1, creatinine measured in all the specimens collected; urine CA19-9 measured in 22 Control, 9 Benign, 47 PDAC. \| Plasma CA19-9 measured in all the specimens collected; LYVE1, REG1B, TFF1 measured in 10 Control, 10 Benign, 14 PDAC. \| \| **Analysis Plan** \| \| \| \| A \| **N=306** (79 Controls, 87 Benign, 140 PDAC I-IV):  REG1A vs REG1B performance in detecting PDAC (Kruskal-Wallis test) \|  \| \| B \| **N=590**:  Urine Panel (LYVE1, REG1B, TFF1) performance in detecting PDAC (Kruskal-Wallis test) \|  \| \| C \| **N=590:**  Performance of the Panel in detecting PDAC (ROC curve analysis):  (a) Control vs PDAC I-II; III-IV; I-IV  Training set (50%): n=187; Validation set (50%): n=195  (b) Benign vs PDAC I-II; III-IV; I-IV  Training set (50%): n=209; Validation set (50%): n=198  Calculation of:  -AUC (95% CI)  -SP at fixed SN in validation set for (a)  -SN at fixed SP in validation set for (b) \|  \| \| D \|  \| **N=350**:  Performance of CA19-9 only; Panel only; CA19-9 + Panel in detecting PDAC (ROC curve analysis)  (a) Control vs PDAC I-IIA; I-II; III-IV; I-IV  (b) Benign vs PDAC I-IIA; I-II; III-IV; I-IV  (c) CP vs PDAC I-IIA; I-II; III-IV; I-IV  Calculation of:  -AUC (95% CI)  -SN (95% CI) and SP (95% CI) at optimal cutpoint  -SP at fixed SN >0.85 for (a)  -SN at fixed SP >0.8 for (b) and (c) \| \| E \|  \| **N=350**:  Performance of PancRISK (‘Normal’ or ‘Elevated’) in combination with different CA19-9 cut-offs (37U/mL, 40U/mL, 45U/mL and 60U/mL)  a) Control vs PDAC I-IV  b) Benign vs PDAC I-IV \| \| F \| **N=78** (22 Control, 9 Benign, 47 PDAC):  Urine CA19-9 performance in detecting PDAC (Kruskal-Wallis test) and comparison with plasma CA19-9 (Spearman correlation). \|  \| \| G \|  \| **N=34** (10 Control, 10 Benign, 14 PDAC):  Plasma LYVE1, REG1B, TFF1 performance in detecting PDAC (Kruskal-Wallis test). \| \| H \| **N=64** (from 4 control subjects):  Urine LYVE1, REG1B, TFF1 stability test (Kruskal-Wallis test). \|  \| \| I \| **N=67** (18 PC, 29 RCC, 20 TCC):  Urine LYVE1, REG1B, TFF1 in urinary tract cancers and comparison with PDAC I-II (Kruskal-Wallis test). \|  \| |
| --- | --- | --- | --- | --- | --- | --- | --- | --- | --- | --- | --- | --- | --- | --- | --- | --- | --- | --- | --- | --- | --- | --- | --- | --- | --- | --- | --- | --- | --- | --- | --- | --- | --- | --- | --- | --- | --- | --- | --- | --- | --- | --- | --- | --- | --- | --- | --- | --- | --- | --- | --- | --- | --- | --- | --- | --- |
